# A quadrupolar two-photon fluorescent probe for *in vivo* imaging of amyloid-β plaques†

**DOI:** 10.1039/c6sc00355a

**Published:** 2016-04-07

**Authors:** Cheol Ho Heo, Avik Ranjan Sarkar, Sung Hoon Baik, Tae Sung Jung, Jeong Jin Kim, Hyuk Kang, Inhee Mook-Jung, Hwan Myung Kim

**Affiliations:** a Department of Chemistry, Department of Energy Systems Research, Ajou University Suwon 443-749 Korea kimhm@ajou.ac.kr +82-31-219-1615; b Department of Biochemistry, Biomedical Sciences College of Medicine, Seoul National University Seoul 110-799 Korea inhee@snu.ac.kr

## Abstract

The formation of beta amyloid (Aβ) plaques in specific brain regions is one of the early pathological hallmarks of Alzheimer's disease (AD). To enable the early detection of AD and related applications, a method for real-time, clear 3D visualization of Aβ plaques *in vivo* is highly desirable. Two-photon microscopy (TPM) which utilizes two near-infrared photons is an attractive tool for such applications. However, this technique needs a sensitive and photostable two-photon (TP) probe possessing bright TP exited fluorescence to impart high signal-to-noise (S/N) visualization of Aβ plaques. Herein, we report a quadrupolar TP fluorescent probe (QAD1) having large TP action cross section (*Φδ*_max_ = 420 GM) and its application for *in vivo* TPM imaging of Aβ plaques. This probe, designed with a centrosymmetric D–A–D motif with a cyclic conjugating bridge and solubilizing unit, displays bright TP excited fluorescence, appreciable water solubility, robust photostability, and high sensitivity and selectivity for Aβ plaques. Using the real-time TPM imaging of transgenic 5XFAD mice after intravenous injection of QAD1, we show that this probe readily enters the blood brain barrier and provides high S/N ratio images of individual Aβ plaques *in vivo*. We also used QAD1 in dual-color TPM imaging for 3D visualization of Aβ plaques along with blood vessels and cerebral amyloid angiopathy (CAA) inside living mouse brains. These findings demonstrate that this probe will be useful in biomedical applications including early diagnosis and treatments of AD.

## Abstract 2

A quadrupolar two-photon fluorescent probe for *in vivo* imaging of amyloid-β plaques is reported.

## Graphical abstract

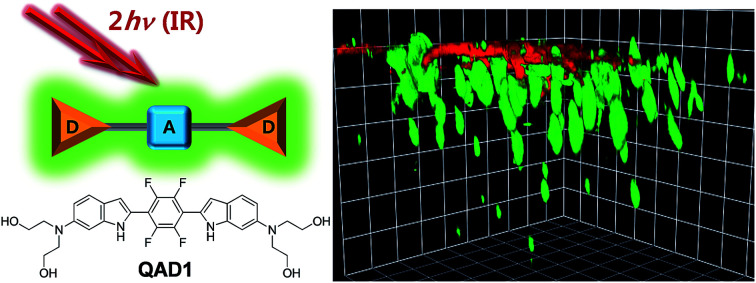

## Introduction

Alzheimer's disease (AD) is a neurodegenerative disorder with chronic dementia and cognitive decline.^1^ The pathological hallmarks of AD include misfolded protein aggregates, and imbalanced reactive oxygen species, metal ions, and acetylcholine levels.^2^ Among them, the formation of beta amyloid (Aβ) plaques and neurofibrillary tangles in specific brain regions has been identified as early pathogenesis of AD.^1,2^ In parallel, substantial trials for AD drug development have been devoted to reducing or preventing Aβ species.^3^ However, the underlying mechanism in the formation of Aβ plaques and its role in AD are barely known. To address this in detail, a method for real-time, clear 3D visualization of Aβ plaques *in vivo* is highly desirable.

Two-photon microscopy (TPM) is an attractive tool for such applications. TPM uses two near-IR photons (>700 nm) that have deep tissue imaging and intrinsic sectioning capability.^4^ It provides three-dimensional (3D) visualization deep inside of intact tissues with high spatial resolution, which is essential for noninvasive applications. With the progress of micro-endoscopic and video-rate scanning systems,^5,6^ TPM also has great potential for clinical uses including early diagnosis, monitoring therapy, and precise treatments. However, for practical applications of TPM, the technique should be combined with a sensitive and photostable TP probe possessing bright TP exited fluorescence (TPEF) to impart high signal-to-noise (S/N) visualization.^7^

To visualize Aβ plaques, a variety of small molecule fluorescent probes have been developed,^8,9^ of which bis-styrylbenzene derivatives such as MeO-X04 have often been used for *in vivo* TPM imaging.^10^ However, their uses in TPM are limited by small values of the TP action cross section (*Φδ*_TPA_) represented for TPEF intensity and/or low water solubility.^9*a*^ Further, the open chain system of the conjugating bridge (C=C) in these probes could lead to fast photobleaching due to photochemical instability processes such as photo-isomerization, thereby making long-term imaging impractical.^11^ Hence, there is a critical need to develop a TP probe for *in vivo* imaging of Aβ plaques with larger value of *Φδ*_TPA_, good water solubility and photostability.

**Scheme 1:**
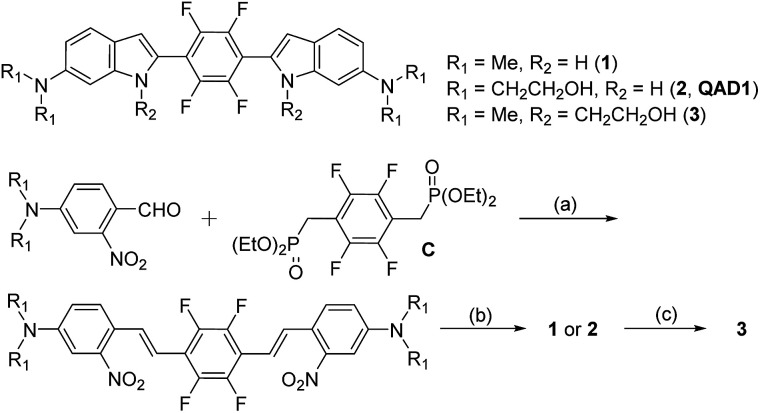
Chemical structures of 1–3 and their synthetic route: *Reagents and conditions*: (a) KO^*t*^Bu, THF, 0–20 °C; (b) P(OEt)_3_, 125 °C; (c) i: compound 1, 2-bromoethylacetate, NaH, 18-Crown-6, THF, 80 °C; ii: NaOMe, CH_3_OH, 0–20 °C.

Toward this end, we designed a quadrupolar TP scaffold with a cyclic conjugating bridge (1, Scheme 1). Regarding the *Φδ*_TPA_ value, the magnitude of TP absorption cross section (*δ*_TPA_) of a molecule is mainly proportional to the extent of intramolecular charge transfer (ICT) character, caused by electron donating and accepting ability, increasing conjugation length, conformational restriction and symmetry, *etc.*^12^ Among various types of TP absorbing dyes, electron donor (D)–acceptor (A) substituted quadrupoles (D–A–D), with a centro-symmetric molecule bearing the quadrupole moment, have been identified as the most promising motif for large *δ*_TPA_ values.^12*b*^ In addition, multi-fluorinated core containing bis-styrylbenzene derivatives have been shown to exhibit selective binding affinity for Aβ plaques.^8*f*^ Therefore, we designed D–A–D type quadrupolar probes for Aβ plaques possessing amino groups as strong electron donors and tetrafluorobenzene as the electron acceptor (1–3, Scheme 1). The C=C bonds were encapsulated within an indolyl cycle with an expectation of higher photostability than the open chain system. We also introduced ethanol as a neutral solubilizing group at the terminal amine (2, QAD1) or the indolyl amine (3) for effective staining to Aβ plaques through the penetration of the blood brain barrier (BBB).

Herein, we report a quadrupole TP probe for Aβ plaques (2, QAD1, Scheme 1) that showed a *Φδ*_TPA_ value larger than 420 GM with resistance to photobleaching, high binding affinity for Aβ plaques, and the ability to penetrate the BBB, thereby allowing real-time, high spatial resolution 3D imaging of Aβ plaques *in vivo* for an extended period of time.

## Results and discussion

Compounds 1 and QAD1 were synthesized by classical Horner–Wadsworth–Emmons coupling reaction between 4-dialkylamino-2-nitrobenzaldehyde and bisphosphonate-substituted tetrafluorobenzene followed by reduction-induced cyclization with P(OEt)_3_ in 65–75% yield (Scheme 1). Compound 3 was prepared by a substitution reaction with compound 1 and 2-bromoethylacetate followed by hydrolysis in 58% yield. The detailed synthetic methods are described in the ESI.†

**Fig. 1:**
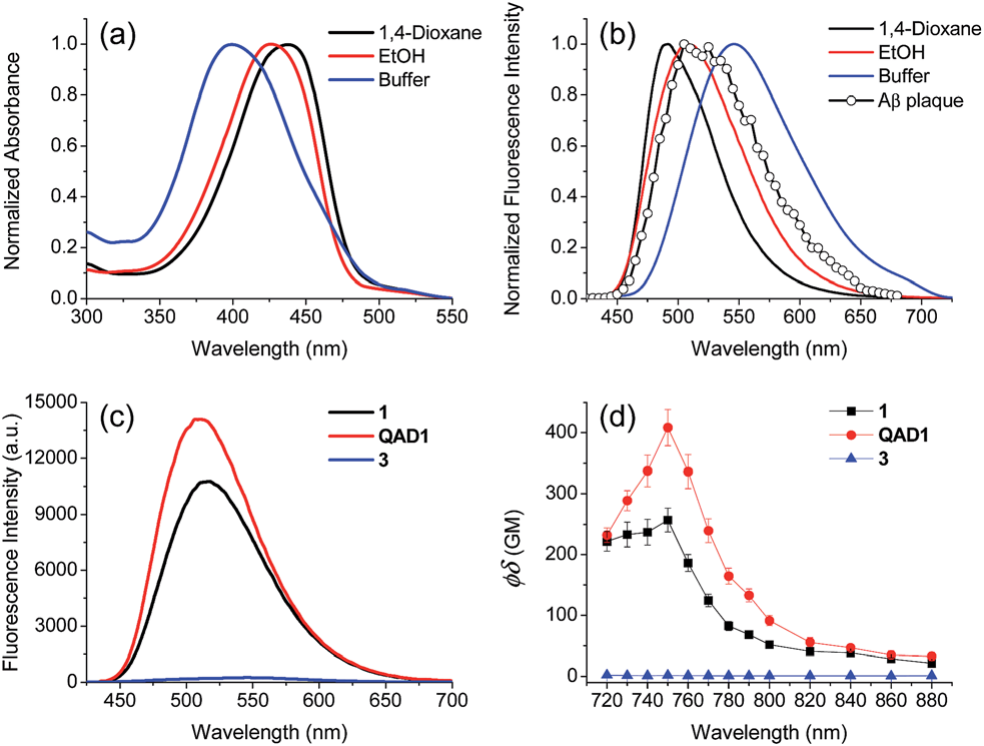
(a) Normalized absorption and (b) emission spectra of QAD1 (1 μM) in 1,4-dioxane, EtOH and PBS buffer (10 mM, pH 7.4). Two-photon excited fluorescence (TPEF) spectra (○) acquired from Aβ plaques in a QAD1-labeled brain slice. (c) Emission spectra of 1, QAD1 and 3 (1 μM) acquired in EtOH. (d) Two-photon action spectra of 1, QAD1 and 3 (1 μM) acquired in EtOH.

First we examined the photophysical properties of 1, QAD1 and 3 in various solvents. The solubility of 1 in phosphate buffer saline (PBS, pH 7.4), determined by a UV-vis titration method, was 1.0 μM, while those for QAD1 and 3 were found to be 4 and 3 μM, respectively (Fig. S1, ESI†), indicating that QAD1 has higher solubility in PBS buffer. Next we tested a sensitivity to the solvent polarity. The emission maximum (*λ*_fl_) of compound 1 was gradually shifted to the red region with increasing solvent polarity from 1,4-dioxane (489 nm) to EtOH (515 nm), whereas no emission was observed in buffer, which might be due to the negligible solubility in buffer (Fig. S2 and Table S1, ESI†). QAD1 showed similar behavior except that the *λ*_fl_ in buffer (10 mM PBS, pH 7.4) appeared in a more red-shifted region (546 nm) than in EtOH (Fig. 1a and b), indicating that QAD1 was a polarity sensitive probe. Further, the *λ*_fl_ of QAD1 in the presence of Aβ aggregates, a good model for the Aβ plaques in aged mouse, was blue-shifted (*λ*_fl_ = 510 nm) from that in PBS buffer, which agreed with the *λ*_fl_ measured in EtOH (Fig. 3a). A similar result was obtained from the QAD1-labeled Aβ plaques in sections of the transgenic mouse brain (508 nm, Fig. 1b), indicating that EtOH can adequately represent the polarity of microenvironment of the Aβ plaques (see below).

Then we characterized the photophysical properties of 1, QAD1 and 3 in EtOH and the results were summarized in Table 1. The one-photon brightness (*εΦ*) of QAD1 was significantly larger than that for 1 (Fig. 1c and Table 1). Interestingly, compound 3 showed dramatically reduced brightness with blue-shifted absorption spectra (Fig. S3, ESI†), although it also displayed a solvatochromic shift (Fig. S2 and Table S1, ESI†).

**Table 1:** Photophysical data for 1, QAD1 and 3^a^

Probe	*λ* ^(1)^_max_ b (10^−4^*ε*)	*λ* ^fl^_max_ c	*Φ* d	*λ* ^(2)^_max_ e	*δ* f	*Φδ* g
1	413 (3.06)	515	0.50	750	540	270
QAD1	426 (4.09)	508	0.73	750	575	420
3	371 (1.46)	521	0.03	720	80	2

a All the measurements were performed in EtOH.

b *λ*_max_ of the one-photon absorption spectra in nm. The numbers in parentheses are molar extinction coefficients in M^−1^ cm^−1^.

c *λ*_max_ of the one-photon emission spectra in nm.

d Fluorescence quantum yield.

e *λ*_max_ of the two-photon excitation spectra in nm.

f Two-photon absorption cross-section in 10^−50^ cm^4^ s per photon (GM).

g Two-photon action cross section in 10^−50^ cm^4^ s per photon (GM).

**Fig. 2:**
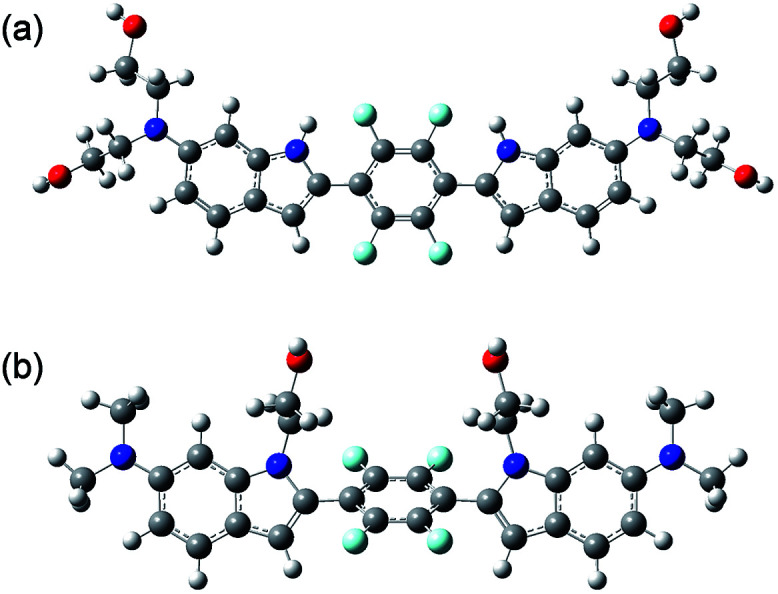
DFT optimized geometries of the *cis*-isomer of (a) QAD1 and (b) 3 in EtOH.

To examine the large difference between QAD1 and 3, we computationally investigated their structures by density functional theory (DFT) at the B3LYP/6-31G* level.^13^ First, the energy difference between the *cis* and *trans*-isomer for QAD1 and 3, respectively, was almost the same (Table S2, ESI†). The optimum geometry for the ground state of QAD1 adopted a planar structure, whereas 3 had a distortion between the central π-core and the bridge heterocyclic ring (Fig. 2). We also investigated the distribution of MOs of the structures of these compounds (Fig. S5, ESI†). The results suggested that QAD1 had a smaller HOMO–LUMO energy (2.56 *vs.* 2.67 eV) with two-fold higher oscillator strength (1.91 *vs.* 0.98) than those for 3. Further, the distortion angle of 3 changed from the ground state (50.8°) to the first electronically excited state (37.7°) that can induce an efficient internal conversion, resulting in fluorescence quenching. Hence, the stronger brightness of QAD1 may be attributed to the planar structure, which can facilitate the effective ICT, whereas the weaker brightness of 3 may be due to the distorted structure, which may cause steric repulsion between the bis-ethanol and the π-core.

**Fig. 3:**
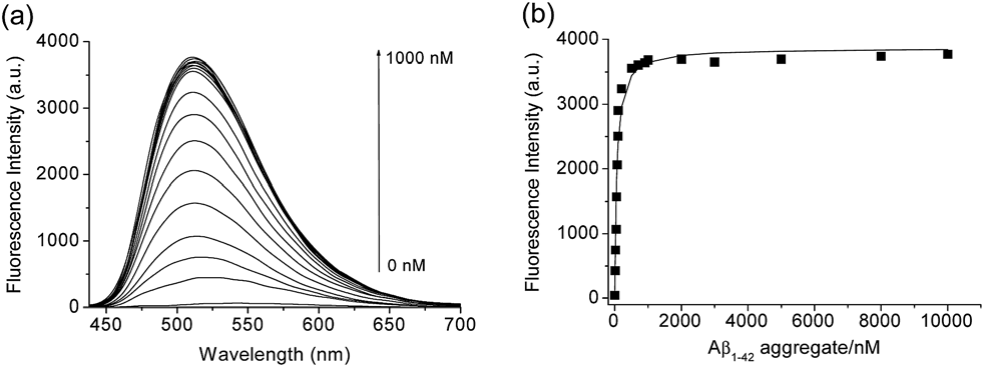
(a) Change in fluorescence intensity and (b) the fluorescence titration curve for the complexation of QAD1 (1 μM) with Aβ_1–42_ aggregates (0–10 μM) in PBS buffer (10 mM, pH 7.4). The calculated value is represented by a solid line. The excitation wavelength was 407 nm and the fluorescence intensity was measured at 510 nm.

We then tested the selective binding profiles of 1, QAD1 and 3 for Aβ aggregates. Upon addition of Aβ aggregates (10 μM) in PBS buffer (10 mM, pH 7.4), the fluorescence intensity of QAD1 (1 μM) increased dramatically (Fig. 3a). The dissociation constant (*K*_d_) value for QAD1/Aβ aggregates was determined by a fluorescence titration method and the value was found to be 16.2 nM, indicating higher binding affinity of QAD1 to Aβ aggregates than those for existing probes (Fig. 3b and Table S3 and S4, ESI†).^8,9^ A similar result was observed with Aβ oligomer (Fig. S6, ESI†), except that the binding affinity for QAD1/Aβ oligomer is slightly decreased (*K*_d_ = 21.5 nM). Moreover, QAD1 had negligible interaction with bovine serum albumin (BSA) and human serum albumin (HSA) under similar experimental conditions (Fig. S7b, ESI†). On the other hand, the increment of emission intensity of 1 in the presence of BSA or HSA was higher than that for Aβ aggregates (Fig. S7a, ESI†), indicating higher affinity for BSA and HSA. A similar result was observed for 3, except for its negligible emission intensity (Fig. S7c, ESI†). Therefore, the specific binding of QAD1 to Aβ aggregates over BSA (or HSA) is likely due to the enhanced solubility and its planar form. Further, the emission intensity of QAD1 was insensitive to pH changes in a biologically relevant pH range (Fig. S4, ESI†). These results revealed that QAD1 is a superior probe for sensitive binding to Aβ aggregates with minimum interference by BSA (or HSA) and pH.

Next, we evaluated the *Φδ*_TPA_ value of QAD1 determined by the TP excited fluorescence (TPEF) method (ESI†). The *Φδ*_max_ value of QAD1 was 420 GM at 750 nm (Fig. 1d and Table 1), which is a larger value than those for dipolar TP probes (∼100 GM).^7^ Moreover, the TPEF intensity of QAD1 in PBS buffer dramatically increased upon binding with Aβ aggregates (Fig. S8, ESI†). The smaller *Φδ*_max_ value of 1 is mainly due to its smaller *Φ* value, and the minimum *Φδ*_max_ value of 3 was likely due to its distorted structure, which may hamper the effective ICT (Table 1). In addition, the lipophilicity value (log *P*_oct_) of QAD1 was 3.42, obtained by partitioning between *n*-octanol and PBS buffer (Table S5, ESI†), which was a well-matched value with an estimated range (log *P* = 2.0–3.5) for penetrating the BBB.^8*c*^ These outcomes suggest that QAD1 would be a sensitive TP probe for bright TPM imaging of Aβ plaques *in vivo*, as we observed (Fig. 6).

**Fig. 4:**
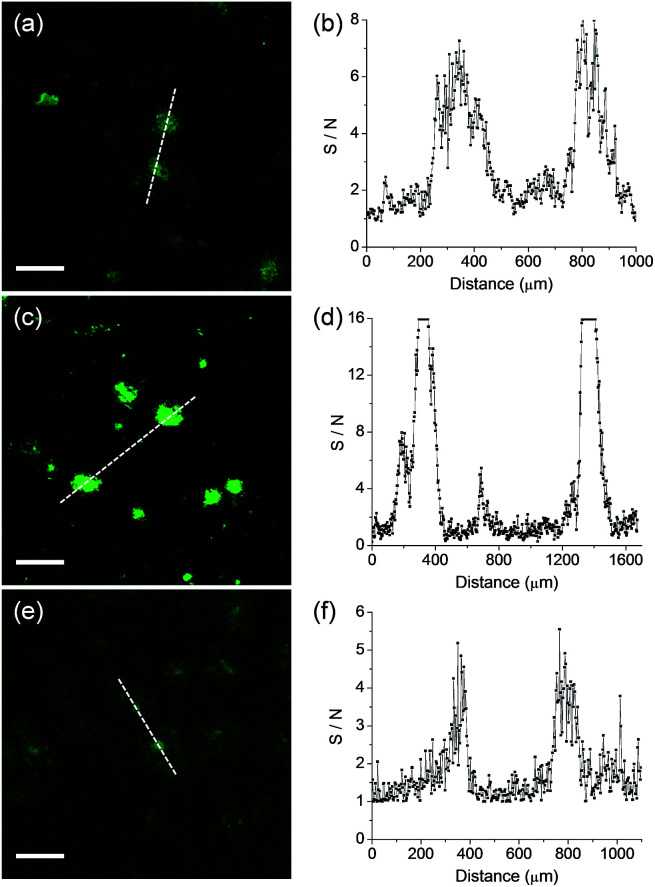
TPM images of a cortical slice of brain from transgenic 5XFAD mice stained with 20 μM (a) 1, (c) QAD1 and (e) 3 for 90 min at 37 °C. (b), (d) and (f): Signal-to-noise (S/N) ratio values measured by TPEF intensity of bright cluster and background regions along the white dotted lines in (a), (c) and (e), respectively. The TPEF intensities were collected at 450–520 nm upon excitation at 750 nm with fs pulse. Scale bars: 48 μm.

We next monitored the ability of QAD1 as a TP probe for Aβ plaques in brain tissues. Cortical slices were taken from a transgenic 5XFAD mouse, an AD model mouse forming Aβ plaques in the brain.^14^ Bright spots in TPM imaging were observed in the QAD1-labeled slice with the highest S/N ratio, while those labeled with 1 and 3 showed dim TPEF with significant background signal (Fig. 4). This observation might be due to the large *Φδ*_TPA_ value and high sensitivity for Aβ plaques of QAD1.

**Fig. 5:**
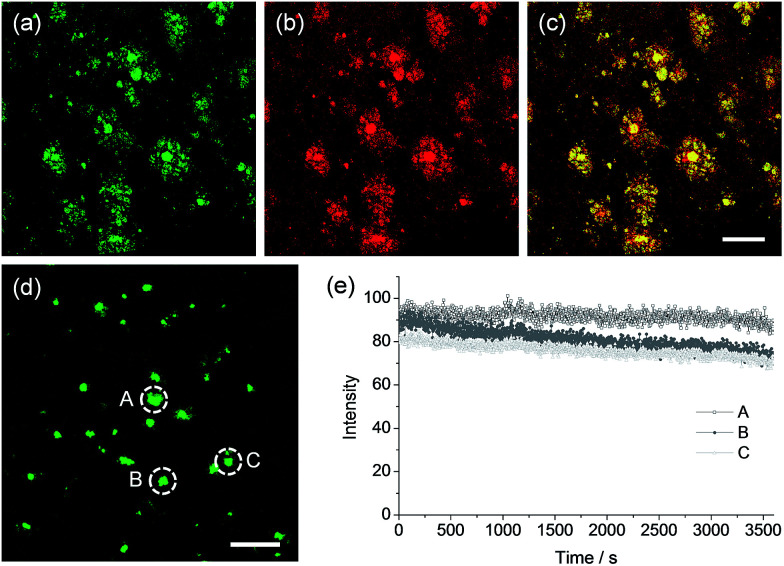
TPM images of cortical slices of brain from transgenic 5XFAD mice co-labeled with (a) QAD1 and (b) Congo red for 90 min at 37 °C, and (c) merged image by 20× magnification. (d) TPM image of a cortical slice of brain from transgenic 5XFAD mice with QAD1. (e) The relative TPEF intensity from A–C in (d) as a function of time. The digitized intensity was continuously recorded with 2.00 s intervals for the duration of 1 h using xyt mode. The TPEF intensities were collected at 450–520 nm upon excitation at 750 nm with femtosecond pulses. Scale bars: (c) 72 and (d) 96 μm.

To confirm whether QAD1 can specifically locate in Aβ plaques, we conducted a co-localization experiment between the probe and Congo red, a well-known fluorescent marker for histology of Aβ plaques.^15^ The bright TPEF regions of QAD1 merged well with signals from Congo red with a Pearson's co-localization coefficient of 0.85, confirming that the bright spots in the TPM images reflected the Aβ plaques (Fig. 5a–c). Here again, the TPEF spectrum from the Aβ plaques in tissue was symmetrical and matched well with the emission spectra acquired in ethanol (Fig. 1b). Moreover, upon excitation at 750 nm with fs pulses, the TPEF intensities of the spots remained nearly the same over 60 min (Fig. 5d and e). This high photostability is probably due to the structural motif of QAD1, encapsulating the C=C bonds within a ring. Further, QAD1 showed high *in vitro* stability in mouse serum as more than 91% intact probe was recognized by HPLC analysis after incubation with mice plasma for 60 min (Fig. S9, ESI†). A cytotoxicity test using MTS assay on human neuronal cell line revealed that QAD1 has low toxicity (Fig. S10, ESI†).

**Fig. 6:**
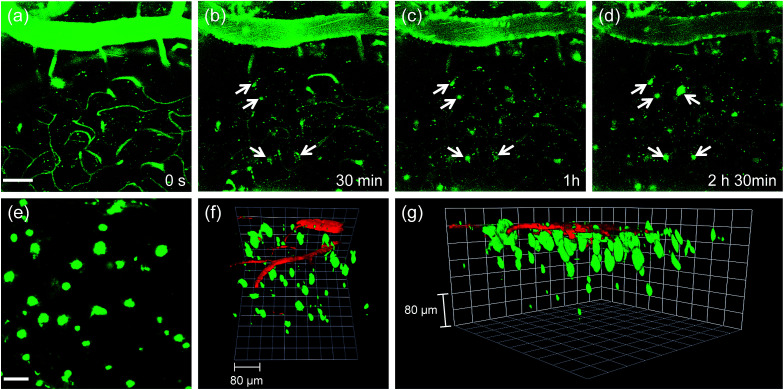
*In vivo* TPM images of the frontal cortex of transgenic 5XFAD mice at (a) 0, (b) 30, (c) 60 and (d) 150 min after i.v. injection of QAD1 (10 mg kg^−1^). (e) 230 sections of TPM images along the *z*-direction at the depth of ∼300 μm from the surface of the cortex were accumulated to visualize Aβ plaques distribution. (f and g) 3D reconstructed TP image of the frontal cortex of transgenic 5XFAD mice after i.v. injection of QAD1 (10 mg kg^−1^) and dextran 40 kDa-Texas red. Approximately 270 sections of TPM images were acquired along the *z*-direction at a depth of ∼300 μm from the surface of the cortex. Scale bars: (a) 50 and (e) 30 μm.

Finally, we tested the utility of QAD1*in vivo*. The transgenic 5XFAD mice were anesthetized and a cranial window was surgically installed for direct TPM imaging. QAD1 (approximately 10 mg kg^−1^) was intravenously injected, then TPM images were immediately collected upon excitation at 780 nm. The initial images showed bright TPEF through the blood vessels in the cortex region (Fig. 6a). The bright intensities at the vessels rapidly decreased with a concomitant increase at the plaques (white arrows in Fig. 6b–d and S11, ESI†). Video S1† clearly visualizes the staining process of QAD1 to the plaques through penetrating of the BBB. Further, kinetic studies revealed that the circulating half-life (*t*_1/2_) calculated by the decay of TPEF intensity at the vessels is 35.7 min (Fig. S11, ESI†). In addition, the TPEF at the plaques appeared within 20 min and reached a peak level in about 2 h, from which the time constants for BBB penetration (*t*_o_ = 23.4 min) and plaque-binding (Δ*τ* = 46.9 min) were calculated by using a sigmoidal Boltzmann equation. Moreover, when TPM images were acquired from regions of greater depth (Fig. 6e), the marked and individual plaques were clearly visualized with high S/N ratio.

Because 3D visualization of Aβ plaques along with blood vessels is important for clinical applications, dextran 40 kDa-Texas red, a well-known blood marker, was injected. Then, dual-color TPM images were immediately obtained along the *z*-direction at depths of more than 300 μm from the surface of the cortex. The 3D images were constructed from approximately 270 sections. As displayed in Video S2† and Fig. 6f and g, individual Aβ plaques of various sizes at the specific positions were clearly visualized along with blood vessels. Furthermore, cerebral amyloid angiopathy (CAA), which is another deposited Aβ form surrounding the wall of blood vessels of the central nervous system,^16^ were directly observed. These outcomes clearly demonstrate the utility of QAD1 for real-time, clear 3D visualization of Aβ plaques *in vivo* and the strong potential for its use in clinical trials for diagnosis and precise treatment of Aβ plaques.

## Conclusions

In summary, we have developed a quadrupole TP probe (QAD1) for *in vivo* imaging of Aβ plaques. This probe shows a large TP action cross section (*Φδ*_max_ = 420 GM), appreciable water solubility, high sensitivity and selectivity for Aβ plaques, high photostability, and easy loading ability into Aβ plaques in living mouse brain. These characteristics allowed direct, 3D visualization of the individual plaques as well as CAA in living mouse brains with high S/N ratio for long periods of time. These results demonstrate that QAD1 will be useful in biomedical applications including early diagnosis and treatments of AD. In addition, this probe provides a good starting point for design of various TP probes with large *Φδ*_max_ value and photostability.

## Experimental sections

### Spectroscopic measurements

Absorption spectra were recorded on a S-3100 UV-Vis spectrophotometer and fluorescence spectra were obtained with FluoroMate FS-2 fluorescence spectrophotometer with a 1 cm standard quartz cell. The fluorescence quantum yield was determined by using coumarin 307 (*Φ* = 0.95 in MeOH) as the reference by the literature method.^17^

### Preparation of Aβ oligomer and aggregates

Aggregated Aβ_1–42_ was prepared according to a literature procedure. For simplification, Aβ_1–42_ protein fragment was purchased from Sigma-Aldrich, which was dissolved in PBS buffer (10 mM, pH 7.4) to make a final concentration of 100 μM. For the preparation of oligomer, the stock solution was allowed to incubate at 4 °C for 24 h with gentle and constant shaking and used for *in vitro* assay directly.^18*a*^ For the aggregation, the stock solution was allowed to incubate at 37 °C for 3 days and used for *in vitro* assay directly.^18*b*^ From this stock solution (oligomer and/or aggregates) different aliquots from 1 nM to 10 μM were added to 1 μM of QAD1 in PBS buffer (10 mM, pH 7.4) followed by gentle shaking for 10 min after addition of each aliquot and their fluorescence intensities were recorded upon excitation at 407 nm. In case of bovine serum albumin (BSA) and human serum albumin (HSA) the stock solutions were prepared to maintain a concentration of 10 μg mL^−1^ in PBS buffer (10 mM, pH 7.4). From this stock solution, 3.0 mL was added to e-tube followed by 3 μL of 1 mM QAD1 in DMSO to maintain a concentration 1 μM of QAD1 in the e-tube and then allowed to gently shake for 1 h at room temperature. Then the solution was transferred into a cuvette and fluorescence intensity was recorded upon excitation at 407 nm.

### Measurement of two-photon cross section

The two-photon cross section (*δ*) was determined by using femtosecond (fs) fluorescence measurement technique as described.^19^ Probes (1.0 × 10^−6^ M) were dissolved in EtOH and the two-photon induced fluorescence intensity was measured at 720–880 nm by using rhodamine 6G as the reference, whose two-photon property has been well characterized in the literature.^20^ The intensities of the two-photon induced fluorescence spectra of the reference and sample emitted at the same excitation wavelength were determined. The TPA cross section was calculated by using the following equation
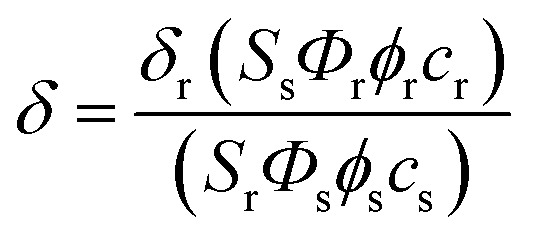
where the subscripts s and r stand for sample and reference molecules. The intensity of the signal collected by a CCD detector was denoted as *S*, *Φ* is the fluorescence quantum yield, and *ϕ* is the overall fluorescence collection efficiency of the experimental apparatus. The number density of the molecules in solution was denoted as *c*, and *δ*_r_ is the TPA cross section of the reference molecule.

### Biostability and cytotoxicity

The biostability of QAD1 was measured according to literature procedure by using mice plasma and HPLC.^8*b*^ In brief, 20 μL of QAD1 (10% ethanol solution, 10 μM) was incubated with 200 μL of mice plasma for 30 and 60 min at 37 °C. Protein were precipitated by addition of acetonitrile (500 μL) after centrifugation at 5000 rpm for 5 min at 4 °C. The liquid phase was collected and then 0.5 mL was taken for HPLC analysis. The eluent was used for the HPLC analysis using acetonitrile–water (80 : 20%) and the flow rate was maintained at 1 mL min^−1^. The UV detector used to perform the analysis had *λ* = 254 nm. The retention time and purity of QAD1 are shown in Fig. S9 (ESI†).

To evaluate the cytotoxicity of QAD1 in SH-SY5Y cells (human neuronal cell line), MTS (cell Titer 96H; Promega, Madison, WI, USA) assay were performed according to the manufacture's protocol. The results are shown in Fig. S10 (ESI†), which revealed that the QAD1 has low cytotoxicity in our incubation condition.

### Animals

For the *in vivo* and *ex vivo* brain imaging study, five familial Alzheimer's disease (5XFAD) transgenic mouse model (The Jackson Laboratory, Bar Harbor, ME) was used. Because 5XFAD mice express three mutations of human amyloid precursor protein (APP) and two mutations of human presenilin1 (PS1) through the neuron-specific promoter, Aβ plaques arise from three months and massive Aβ plaques are detected in the brain of 10 month old mice. Consequently, neuronal cell death and memory loss occurs.^21^ Mice were bred with the 12/12 h light–dark cycle in the specific pathogen-free facility. All animal experiments were followed and approved by the Ethics Review Committee for Animal Experimentation of Seoul National University.

### Animal surgery

We performed open skull craniotomy surgery for the intravital two-photon imaging as previously described.^22^ Briefly, the mouse was anesthetized with intramuscular (i.m.) injection of Zoletil 50 (2 mL kg^−1^; Virbac, Carros, France)-Rompun (25%; Bayer Korea, Seoul, South Korea) mixture and the head was fixed on the customized heating plate (Live Cell Instrument, Seoul, South Korea) to maintain body temperature (37 °C) during the surgery and the imaging. After removal of scalp and periosteum, the skull region especially between −0.5 and −3.5 mm relative to bregma and 0.5 to 3.5 mm relative to sagittal suture was taken apart with drilling and the exposed region was covered with a 3 mm round coverslip. QAD1 was intravenously (i.v.) injected (10 mg kg^−1^) just before imaging.

### Two-photon fluorescence microscopy

Two-photon fluorescence microscopy images of 1, QAD1 and 3 labeled tissues were obtained with spectral confocal and multiphoton microscopes (Leica TCS SP8 MP) with ×10 dry, ×40 oil and ×100 oil objectives, numerical aperture (NA) = 0.30, 1.30 and 1.30. The two-photon fluorescence microscopy images were obtained with a DMI6000B Microscope (Leica) by exciting the probes with a mode-locked titanium–sapphire laser source (Mai Tai HP; Spectra Physics, 80 MHz, 100 fs) set at wavelength 750 nm and output power 2680 mW, which corresponded to approximately 3.0 mW average power in the focal plane. Live imaging was performed using the incubator systems (Chamlide IC; Live Cell Instrument) for stable imaging environment by maintaining appropriate temperature, humidity and pH over the long term. To obtain images at 450–520 nm internal PMTs were used to collect the signals in an 8 bit unsigned 512 × 512 and 1024 × 1024 pixels at 400 Hz scan speed, respectively.

To visualize QAD1 amyloid plaques staining *in vivo*, using LSM 7 MP two-photon laser scanning microscopy (Carl Zeiss Inc., Oberkochen, Germany) and titanium–sapphire femtosecond laser (Chameleon Ultra, Coherent, Santa Clara, CA), *in vivo* two-photon brain imaging was performed. Laser power was set to 30–50 mW and 780 nm wavelength was used for the brain imaging. For the deep tissue z-stack imaging, 1 μm interval was adjusted and 4D imaging was obtained by exploiting time-lapse z-stack imaging. Volocity (PerkinElmer, Boston, MA) software was used for rendering.

### Cortical slice preparation for *ex vivo* two photon imaging

Cortical slices were prepared from transgenic 5XFAD mice. 5XFAD mice were sacrificed with cervical dislocation and the whole brain was rapidly removed from the cranium and placed for 30 s in ice-cold artificial cerebrospinal fluid (aCSF) containing NaCl (124 mM), KCl (3 mM), NaH_2_PO_4_ (1.25 mM), MgCl_2_ (1 mM), NaHCO_3_ (36 mM), d-glucose (10 mM), CaCl_2_ (2 mM) and bubbled with 95% O_2_/5% CO_2_. Coronal slices were cut into 400 μm thicknesses using a vibrating-blade microtome in aCSF. Slices were incubated with 2 μL of 10 mM stock solution of 1, QAD1 and 3 in DMSO (total 20 μM) in aCSF bubbled with 95% O_2_ and 5% CO_2_ for 1 h 30 min at 37 °C. Slices were then washed three times with aCSF and transferred to glass-bottomed dishes (NEST) and observed in a spectral confocal multiphoton microscope.

## Supplementary Material

SC-007-C6SC00355A-s001

SC-007-C6SC00355A-s002

SC-007-C6SC00355A-s003
