# Supplementary material for: A quadrupolar two-photon fluorescent probe for in vivo imaging of amyloid-β plaques
Source: Chem Sci. 2016 Apr 7;7(7):4600–6. doi: 10.1039/c6sc00355a (PMC6016450; doi:10.1039/c6sc00355a)
Supplement: SC-007-C6SC00355A-s001 [file SC-007-C6SC00355A-s001.pdf]

Electronic Supplementary Material (ESI) for Chemical Science.
This journal is © The Royal Society of Chemistry 2016

Supporting Information to Accompany

"A Quadrupolar Two-Photon Fluorescent Probe for *In Vivo* Imaging of Amyloid-β Plaques"

Cheol Ho Heo,[a] Avik Ranjan Sarkar,[a] Sung Hoon Baik,[b] Tae Sung Jung,[a] Jeong Jin Kim,[a] Hyuk Kang,[a] Inhee Mook-Jung*[b] and Hwan Myung Kim*[a]

[a]Department of Chemistry and Department of Energy Systems Research, Ajou University, Suwon 443-749, Korea, [b]Department of Biochemistry and Biomedical Sciences College of Medicine, Seoul National University, Seoul, 110-799, Korea.

Table of Contents

	Page
**Synthesis of 1**, **2 (QAD1)** and **3.**	S3
**Water Solubility.**	S9
**Octanol-water partition coefficient (log P_{oct}).**	S15
**Fig. S1** (a,c,e) One-photon absorption spectra and (b,d,f) plot of intensity against probe concentration for (a,b) **1**, (c,d) **QAD1** and (e,f) **3** in PBS buffer (10mM, pH 7.4).	S9
**Fig. S2** (a, c, e) Normalized absorption and (b, d, f) emission spectra of (a, b) **1**, (c, d) **QAD1** and (e, f) **3** and in 1,4-dioxane, EtOH, EtOH: PBS (v/v, 1:1) and PBS buffer (pH 7.4).	S10
**Fig. S3** Molar absorptivity spectra of **1**, **QAD1** and **3** (1 μM) acquired in EtOH.	S11
**Fig. S4** Effect of pH for **QAD1** in universal buffer (0.1 M citric acid, 0.1 M KH_2PO_4, 0.1 M $Na_2B_4O_7$, 0.1 M Tris, 0.1 M KCl). The excitation wavelength was 407 nm.	S12
**Fig. S5** DFT (Density Functional Theory) optimized geometries and MO's distribution of (a) **QAD1** and (b) **3** in EtOH.	S13
**Fig S6.** (a) Change in emission intensity and (b) the fluorescence titration curve for the complexation of **QAD1** (1 μM) with Aβ_{1-42} oligomer (0-10 μM) in PBS buffer (10 mM, pH 7.4). The calculated value is represented by solid line. The excitation wavelength was 407 nm and the fluorescence intensity was measured at 517 nm.	S14
**Fig. S7** The change in emission intensity (a) **1**, (b) **QAD1** and (c) **3** in presence of Aβ_{1-42} aggregated, Aβ_{1-42} oligomer, BSA and HSA in PBS buffer (10 mM, pH 7.4). (d) Relative fluorescence intensity upon addition of particular protein in PBS (10 mM, pH 7.4) measured at 504, 510 and 506 nm for **1**, **QAD1** and **3**, respectively. The excitation wavelengths were 417 nm for **1**, 407 nm for **QAD1** and 326 nm for **3**, respectively.	S16
**Fig. S8** Two-photon fluorescence response of 1 μM **QAD1** in the presence of Aβ_{1-42} oligomer	S17

(10 μM) and Aβ_{1-42} aggregates (10 μM) in PBS buffer (10 mM, pH 7.4). The excitation wavelength was 750 nm.
Fig. S9 The HPLC profile of **QAD1** (a) before and (b,c) after incubation with mice plasma for (b) 30 and (c) 60 min. (d) The HPLC profile of mice plasma. S18
Fig. S10 Viability of SH-SY5Y cells in the presence of **QAD1** as measured by using MTS assay. The cells were incubated with (a) 0–20 μM **QAD1** for 12 h and (b) 20 μM **QAD1** for 2, 6 and 12 h. (n = 6) S18
Fig. S11 *In Vivo* TPM images of the frontal cortex of transgenic 5XFAD mice at (a) 0, (b) 30, (c) 60 and (d) 150 min after i.v. injection of **QAD1** (10mg/Kg). Scale bar: 50 μm. (e) Two-photon excited fluorescence intensity (normalized) as a function of time acquired from the blood vessels (red circle in Fig S11 a-d) and the plaque (white circle in Fig S11 a-d). (f) Decay rates of the TPEF in the blood vessels (red circle). The slopes of these plots are the 1st order decay constant. Half-life ($t_{1/2}$) were calculated by using the relationship, $t_{1/2} = 0.693/k$. S19
Fig. S12 ^{1}H-NMR spectrum (400 MHz) of **1** in $CDCl_3$. S20
Fig. S13 ^{13}C-NMR spectrum (100 MHz) of **1** in $CDCl_3$ containing 2 drops of d_6-DMSO. S20
Fig. S14 HRMS spectrum of **1.** S21
Fig. S15 ^{1}H-NMR spectrum (400 MHz) of **2** (**QAD1**) in d_6-DMSO. S21
Fig. S16 ^{13}C-NMR spectrum (100 MHz) of **2** (**QAD1**) in d_6-DMSO. S22
Fig. S17 HRMS spectrum of **2** (**QAD1**)**.** S22
Fig. S18 ^{1}H-NMR spectrum (400 MHz) of **3** in $CDCl_3$. S23
Fig. S19 ^{13}C-NMR spectrum (100 MHz) of **3** in $CDCl_3$ containing 2 drops of d_6-DMSO. S23
Fig. S20 HRMS spectrum of **3.** S24
Table S1. Photophysical data for **1**, **QAD1** and **3** in various solvents. S11
Table S2. DFT (Density functional theory) calculation of **QAD1** and **3** in EtOH. S13
Table S3. Complexation Study of **QAD1** with Aβ_{1-42} aggregated and oligomer. S14
Table S4. Photophysical data of two-photon imaging probes for Aβ plaques. S15
Table S5. Molar extinction coefficients and logP_{oct} for listed probes in *n*-octanol and PBS. S17

Synthesis of 1, 2 (QAD1) and 3. Compound **C**[1] was prepared by the literature method and the syntheses of **1-3** were accomplished according to following schemes S1-S3. The synthetic procedures of intermediates, **1-3** are described below.

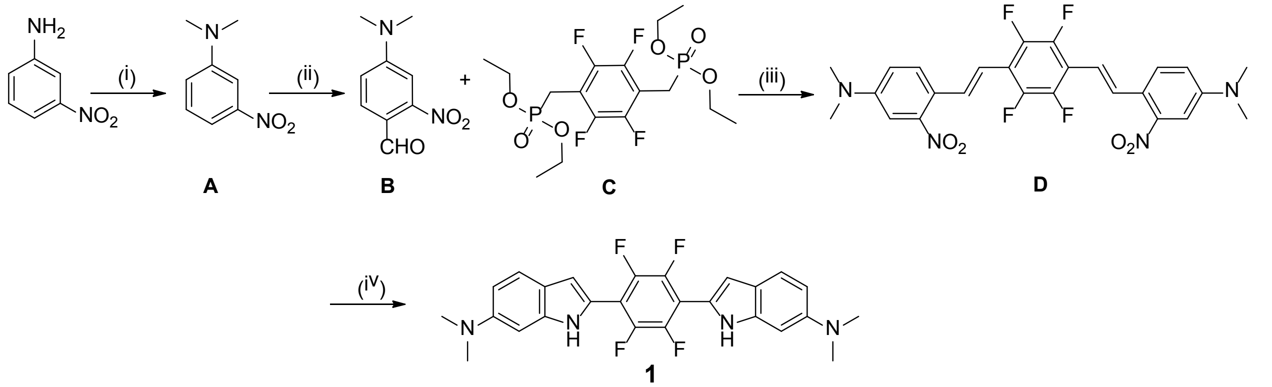

Scheme S1. Reagents and conditions: (i) Iodomethane, proton-sponge, CH_3CN, reflux, 16hr; (ii) Phosphorous oxychloride ($POCl_3$), DMF, 90 ^{0} C, 8hr; (iii) Potassium tert-butoxide, THF, 0 ^{0}C to rt, 12hr; (iv) Triethyl phosphite ($P(OEt)_3$), 125 ^{0}C, 12hr.

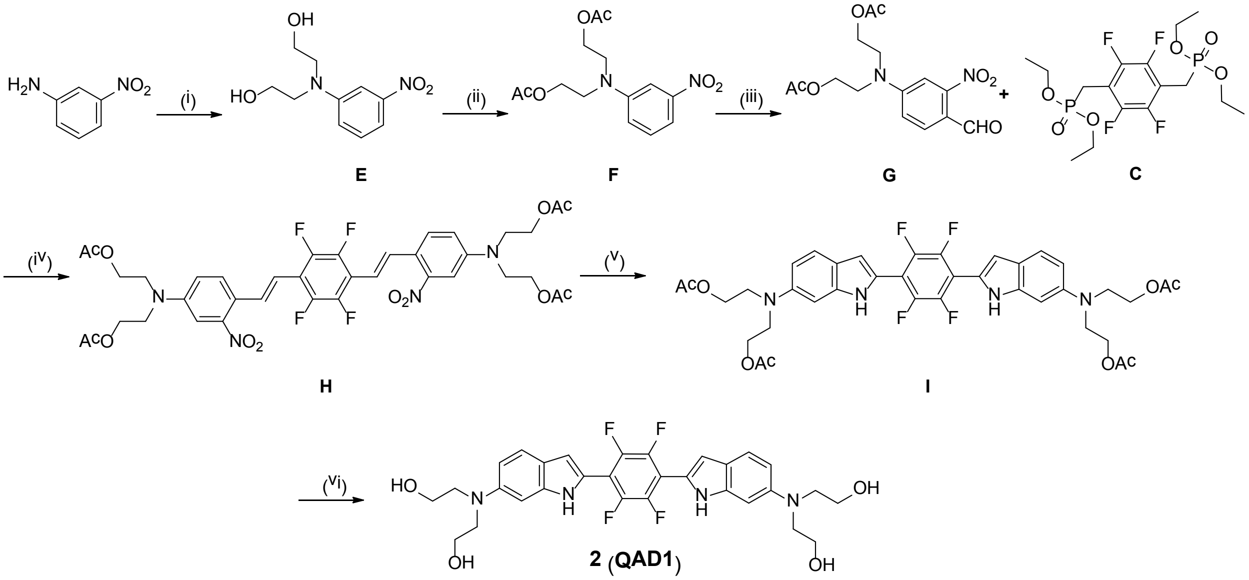

Scheme S2. Reagents and conditions: (i) 2-Bromoethanol, calcium carbonate, potassium iodide, H_2O, 100 ^{0}C, 8 hr; (ii) Acetyl chloride, triethylamine, 35 ^{0}C, 12hr; (iii) Phosphorous oxychloride, DMF, 90 ^{0}C, 12hr; (iv) potassium tert-butoxide, THF, 0 ^{0}C to rt, 12hr; (v) Triethyl phosphite ($P(OEt)_3$), 125 ^{0}C, 12hr; (vi) NaOMe, CH_3OH, 0 ^{0}C to rt, 12hr.

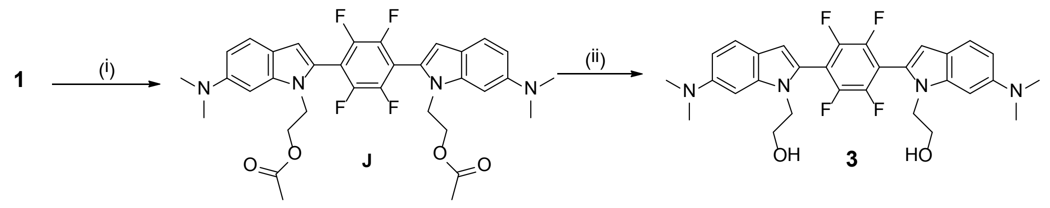

Scheme S3. Reagents and conditions: (i) 2-bromoethylacetate, NaH, 18-Crown-6, THF, 80 ^{0}C to rt; (ii) NaOMe, CH_3OH.

Compound A. To a stirring solution of 3-nitro aniline (2.0 g, 14.5 mmol) and proton-sponge (6.83 g, 31.86 mmol) in anhydrous CH_3CN (70 mL), iodomethane (2.0 mL, 31.9 mmol) was added. The whole reaction mass was allowed to stirring at 80 ^{0}C for 8h under nitrogen atmosphere. The solvent was evaporated to obtain the residue which was dissolved in $CHCl_3$ (100 mL) and washed with water (100 mL) and 1(N) H_2SO_4 (50 mL) subsequently. The organic portion was dried over Na_2SO_4 and distilled out the organic solvent to obtain the crude which was purified by column chromatography (2:8 ethyl acetate: hexanes as an eluent) afforded **A** as a red solid. Yield: 1.51 g (63 %). ^{1}H NMR (400MHz, $CDCl_3$): δ (ppm) 7.52-7.47 (m, 2H), 7.32 (t, J = 8.0 Hz, 1H), 6.95 (dd, J = 8.8 Hz, J = 2.8 Hz, 1H), 3.03 (s, 6H). ^{13}C NMR (100 MHz, $CDCl_3$): δ (ppm) 150.4, 148.9, 129.3, 117.4, 110.2, 105.7, 40.2.

Compound B. To a solution of **A** (1.3 g, 7.83 mmol) in anhydrous DMF (2.17 mL, 28.27 mmol) in two-necks round-bottomed was cooled to 0 ^{0}C in an ice bath and $POCl_3$ (1.07 mL, 11.74 mmol) was added dropwise. Then the reaction mixture was heated within 1 hr. to 80 ^{0}C and stirred at this temperature for additional 6hr. The dark solution then cooled to room temperature and poured into ice cooled water and the precipitate obtained was filtered off. The residue was recrystallized from acetone to afford compound **B** as a brown solid. (1.2 g, 79%). ^{1}H NMR (400 MHz, $CDCl_3$): δ (ppm) 10.12 (s, 1H), 7.91 (d, J = 2.8 Hz, 1H), 7.09 (d, J = 2.4 Hz, 1 H), 6.84 (dd, J = 8.8 Hz, J = 2.0 Hz, 1H), 3.14 (s, 6 H) ppm; ^{13}C NMR (100 MHz, $CDCl_3$): δ (ppm) 186.5, 154.2, 150.9, 131.5, 117.3, 114.5, 105.9, 40.3.

Compound D. To a stirred solution of **C** (1.0 g, 2.33 mmol) in anhydrous THF (80 mL), NaO*t*Bu (0.654 g, 5.83 mmol) was added at 0 ^{0}C under N_2 atmosphere. After 30 min, **B** (0.95 g, 4.89 mmol) in

THF (20 mL) was added to the reaction mass and stirred for overnight at rt. Water was added to quench the reaction and red precipitate appear, collect the solid residue and washed with THF followed by water and hexane. The residue dried in vacuo to obtain the **D** (0.730 g, 63%) as a red solid. ^{1}H NMR (400 MHz, $CDCl_3$): δ (ppm) 7.86 (d, *J* =16 Hz, 1H), 7.64 (d, *J* =8.0 Hz, 1H), 7.19 (s, 1 H), 6.92 – 6.87 (m, 2H), 3.07 (s, 6 H).

Compound 1. A solution of **D** (0.05 g, 0.094 mmol) in $P(OEt)_3$ (5 mL) was heated under reflux overnight under nitrogen atmosphere. The solvent was removed in vacuo and the product was purified on a silica column by using 3:7 ethyl acetate: hexane to yield the compound **1** (0.032 g, 72%) as a brick red solid. ^{1}H NMR (400 MHz, $CDCl_3$): δ ppm 9.12 (s, 1H), 7.40 (d, *J* = 8.8 Hz, 1 H), 6.97 (s, 1H), 6.69-6.64 (m, 2H), 2.90 (s, 6 H). ^{13}C NMR (100 MHz,$CDCl_3$ contained 2-drops of d_6-DMSO): δ (ppm) 148.5, 144.7, 142.3 (d, *J* = 10.7Hz), 138.2, 122.2, 121.1, 119.7, 110.1, 106.8, 93.5, 41.4. HRMS (FAB^+): m/z calcd for [$C_{26}H_{22}N_4F_4$]: 466.1775, found: 466.1776

Compound E. 3-nitro aniline (10.0 g, 72.4 mmol), 2-bromoethanol (12.14 mL, 181.0 mmol), $CaCO_3$ (18.11 g, 181.0 mmol) and KI (1.2 g, 7.2 mmol) were added to 150 mL water and refluxed for 16 h. After filtration, the filtrate was extracted with EtOAc (50 mL x 3), and the combined organic layers were further washed with saturated NaCl (50 mL x 2), dried over anhydrous Na_2SO_4, filtered and concentrated to obtain the crude. The crude product was purified by silica column chromatography by using 7:3 ethyl acetate: hexane as an eluent to afford the desired product **E** as a yellow solid. Yield: 6.20 g (38 %). ^{1}H NMR (400MHz, $CDCl_3$): δ (ppm) 7.36-7.35 (m, 2H), 7.16 (t, *J* = 8.0 Hz, 1H), 6.95 (dd, *J* = 8.4 Hz, *J* = 2.8 Hz, 1H), 4.68 (t, *J* = 4.8 Hz, 2H), 3.69-3.65 (m, 4H), 3.47 (t, *J* = 5.6 Hz, 4H). ^{13}C NMR (100 MHz, $CDCl_3$): δ (ppm) 148.9, 148.6, 129.3, 117.7, 110.2, 105.8, 59.4, 54.8.

Compound F. A solution **E** (5.4 g, 23.87 mmol) and triethylamine (7.33 mL, 52.52 mmol) in two necks round-bottomed flux was cooled to 0 ^{0}C. Acetyl chloride (3.73 mL, 52.52 mmol) was then added drop wise and the solution was stirred overnight at 35 ^{0}C. After cooling down to room temperature, the mixture was hydrolyzed with 25 mL of water. After vigorous stirring for 1h, the THF was evaporated

and saturated aqueous solution of $NaHCO_3$ was used to neutralize. After extraction with chloroform (3 x 50 mL), the combined organic layers was dried over Na_2SO_4 and filtered, and the solvent was removed under vacuum to obtained the compound **F** as a yellow solid (7.0 g, 95%). ^{1}H NMR (400 MHz, $CDCl_3$): δ (ppm) 7.45 (t, *J* = 2.8 Hz, 1H), 7.32 (dd, *J* = 8.0 Hz, *J* = 2.0 Hz, 1 H), 7.17 (t, *J* = 8 Hz, 1H), 6.94 (dd, *J* = 8.4 Hz, *J* = 6.0 Hz, 1 H), 4.12 (t, *J* = 6.0 Hz, 4 H), 3.56 (t, *J* = 6.4 Hz, 4 H), 1.88 (s, 6 H). ^{13}C NMR (100 MHz, $CDCl_3$): δ (ppm) 170.2, 148.9, 147.8, 129.5, 117.1, 110.7, 105.7, 60.8, 49.3, 20.4.

Compound G. Freshly distilled DMF (3.10 mL, 32.22 mmol) in two-necks round-bottomed was cooled to 0 ^{0}C in an ice bath and $POCl_3$ (1.62 mL, 17.72 mmol) was added dropwise. After stirring for 1h at this temperature, the solution of **F** (5.0 g, 16.11 mmol) in DMF was then added and the reaction mass was stirred at 70 ^{0}C for overnight. The dark solution then cooled to room temperature and poured into ice cooled water. A saturated solution of sodium acetate was slowly added and mixture was stirred overnight. After overnight stirring brown precipitate was appeared and collect the precipitate and washed with acetone to obtain the compound **G** (2.80 g, 52%) as a deep brown solid. ^{1}H NMR (400 MHz, $CDCl_3$): δ (ppm) 10.09 (s, 1H), 7.86 (d, *J* = 8.8 Hz, 1H), 7.28 (d, *J* = 5.6 Hz, 1 H), 6.98 (dd, *J* = 8.8, *J* = 2.8 Hz, 1 H), 4.27 (t, *J* = 5.6 Hz , 4 H), 3.74 (t, *J* = 5.6 Hz, 4 H), 2.03 (s, 6 H). ^{13}C NMR (100 MHz, $CDCl_3$): δ (ppm) 186.2, 170.6, 152.4, 151.8, 131.5, 118.4, 114.7, 106.5, 60.8, 49.8, 20.9.

Compound H. To a stirred solution of **C** (0.5 g, 1.11 mmol) in anhydrous THF (25 mL), NaO*t*Bu (0.274 g, 2.44 mmol) was added at 0 ^{0}C under N_2 atmosphere. After 30 min, **G** (0.826 g, 2.44 mmol) in anhydrous THF (20 mL) was added to the reaction mass and stirred for overnight at rt. Water was then added to quenched the reaction and red precipitate appear, collect the precipitate and washed with water followed by hexane, crude product was obtained. The crude product was purified by silica column chromatography by using 7:3 ethyl acetate: hexane to get the compound **H** as a red solid (0.205 g, 25%). ^{1}H NMR (400 MHz, $CDCl_3$): δ (ppm) 7.83 (d, *J* = 16.8 Hz, 1 H), 7.64 (d, *J* = 9.2 Hz, 1 H), 7.34 (d, *J* = 2.4 Hz, 1 H), 7.02 (dd, *J* = 9.2, *J* = 5.6 Hz, 1 H), 6.88 (d, *J* = 16.8 Hz, 1 H), 4.28 (t, *J* = 5.6

Hz, 4H), 3.70 (t, *J* = 5.6 Hz, 4H), 2.05 (s, 6 H) ppm. ^{13}C NMR (100 MHz, $CDCl_3$): δ (ppm) 170.7, 149.4, 147.9, 145.9, 143.3, 131.7, 128.9, 120.2, 116.3, 115.2, 107.3, 61.2, 49.8, 21.1.

Compound I. A solution of **H** (0.08 g, 0.098 mmol) in $P(OEt)_3$ (5 mL) was heated under reflux at 125 ^0C for overnight under N_2 atmosphere. The solvent was removed in vacuo and the product was purified on a silica column chromatography using 7:3 ethyl acetate: hexane to pure ethyl acetate to yield the compound **I** (0.050 g, 68%) as a red solid. ^1H NMR (400 MHz,$CDCl_3$ contained 2-drops of d_6-DMSO): δ (ppm) 9.31 (s, 1H), 7.34 (d, *J* = 8.8 Hz, 1 H), 6.89 (s, 1H), 6.59 (s, 1H), 6.58 (dd, *J* = 8.8 Hz, *J* = 1.6 Hz, 1 H), 4.15 (t, *J* = 6.0 Hz, 4H), 3.54 (t, *J* = 6.0 Hz, 4H), 1.93 (s, 6H). ^{13}C NMR (100 MHz, $CDCl_3$ contained 2-drops of d_6-DMSO): δ (ppm) 170.5, 144.6, 142.2, 138.5, 138.3, 122.3, 121.5, 119.9, 108.9, 106.5, 93.3, 61.4, 50.3, 20.8.

Compound 2 (QAD1). A solution of **I** (0.05 g, 0.026 mmol) in methanol (10 mL) was added 20 μL of NaOMe solution and stirred the reaction mass at 0 ^0C for 60 min. Then the solvent was evaporated and the residue was dissolved in $CHCl_3$ (25 mL, contained 2.5 mL MeOH) washed with saturated NH_4Cl solution (25 mL). The organic layer was dried over anhydrous $MgSO_4$ and concentrated under *in vacuo*. The crude product was further purified by silica column chromatography using 1:20 CH_3OH:$CHCl_3$ as an eluent to obtain **2** as a deep brown solid (quantitative yield). ^1H NMR (300 MHz, d_6-DMSO): δ (ppm) 10.89 (s, 1H), 7.41 (d, *J* = 9.2 Hz, 1 H), 6.85 (s, 1H), 6.73 (s, 1 H), 6.57 (d, *J* = 8.0 Hz, 1 H), 4.78 (bs, 2H), 3.59 (t, *J* = 6.0 Hz, 4H), 3.48 (t, *J* = 6 .4 Hz, 4H). ^{13}C NMR (100 MHz, d_6-DMSO): δ (ppm) 145.3, 139.1, 127.8, 124.5, 122.7, 120.8, 120.4, 118.7, 109.6, 108.6, 58.3, 53.9. HRMS (FAB^+): m/z calcd for [$C_{30}H_{30}N_4O_4F_4$]: 586.2198, found: 586.2198

Compound J. NaH (0.016 g, 0.68 mmol) and 18-Crown-6 (0.011 g, 0.045 mmol) were dissolved in anhydrous THF (10 mL) under nitrogen atmosphere and allowed to stirring for 10 min. Then a solution of compound **2** (0.07 g, 0.15 mmol) in THF was added dropwise to the reaction mass and allowed to reflux for 1hr at 80 ^0C. After 1hr the reaction mass was allowed to stirring at rt then 0 ^0C, at this temperature a solution of 2-bromoethylacetate (36 μL, 0.33 mmol) in THF was added dropwise and

allowed to stirring overnight at rt. After completion of the reaction THF was distilled out *in vacuo* and the residue was dissolved in ethylacetate (50 mL) and washed with water (2 x 25 mL), the organic layer dried over Na_2SO_4 and distilled out the organic solvent to obtain the crude which was purified by column chromatography (3:7 ethyl acetate: hexanes as an eluent) afforded **J** as a yellow solid. Yield: 0.067 (71 %). ^{1}H NMR (400MHz, $CDCl_3$): δ (ppm) 7.55 (d, *J* = 8.8 Hz, 1H), 6.84 (dd, *J* = 8.8 Hz, *J* = 2.4 Hz, 1 H), 6.70 (d, *J* = 1.6 Hz, 1H), 6.50 (s, 1H), 4.30-4.27 (m, 4H), 3.04 (s, 6H), 1.92 (s, 3H). ^{13}C NMR (100 MHz, $CDCl_3$): δ (ppm) 170.6, 148.5, 145.9, 143.3, 139.1, 122.2, 121.7, 120.3, 113.2, 110.2, 106.7, 93.1, 64.4, 41.9, 29.9, 20.8.

Compound 3. A solution of **J** (0.05 g, 0.078 mmol) in methanol (10 mL) was added 20 μL of NaOMe solution and stirred the reaction mixture at 0 ^{0}C for 60 min. Then the solvent was evaporated and the residue was dissolved in $CHCl_3$ (25 mL, contained 2.5 mL MeOH) washed with saturated NH_4Cl solution (25 mL). The organic layer was dried over anhydrous $MgSO_4$ and concentrated under *vacuo*. The crude was purified by silica column chromatography using 7:3 ethyl acetate: hexanes as an eluent to obtain **3** as a yellow solid. Yield: 0.034 g (81 %). ^{1}H NMR (300 MHz, $CDCl_3$): δ (ppm) 7.55 (d, *J* = 8.4 Hz, 1 H), 6.83 (dd, *J* = 8.8 Hz, *J* = 2.0 Hz, 1 H), 6.68 (d, *J* = 2.0 Hz, 1 H), 6.63 (s, 1 H), 4.21 (t, *J* = 5.6 Hz, 2H), 4.84 (t, *J* = 5.2 Hz, 2H). ^{13}C NMR (100 MHz, $CDCl_3$ contained 2-drops of d_6-DMSO): δ (ppm) 145.5, 138.9, 132.6, 122.6, 121.4, 120.2, 113.4, 109.9, 106.3, 93.4, 57.8, 42.8, 29.8. HRMS (FAB^+): m/z calcd for [$C_{30}H_{30}N_4O_2F_4$]: 554.2299, found: 554.2297.

Water Solubility of QAD1. Small amount of dye was dissolved in DMSO to prepare the stock solutions (1.0×10^{-2} M). The solution was diluted to ($1.0 \times 10^{-5} \sim 1.0 \times 10^{-7}$) M and added to a cuvette containing 3.0 mL of PBS buffer (10 mM, pH 7.4) by using a micro syringe. In all cases, the concentration of DMSO in H_2O was maintained to be 0.1 %.[2] The plot of absorption intensity against the total amount of the dye injected to the cuvette was linear at low dye content and showed curvature as more dye was added. The maximum point in the linear region was taken as the solubility. The solubility of **1**, **QAD1** and **3** in PBS buffer were ~1.0, ~4.0 and ~3.0 μM, respectively.

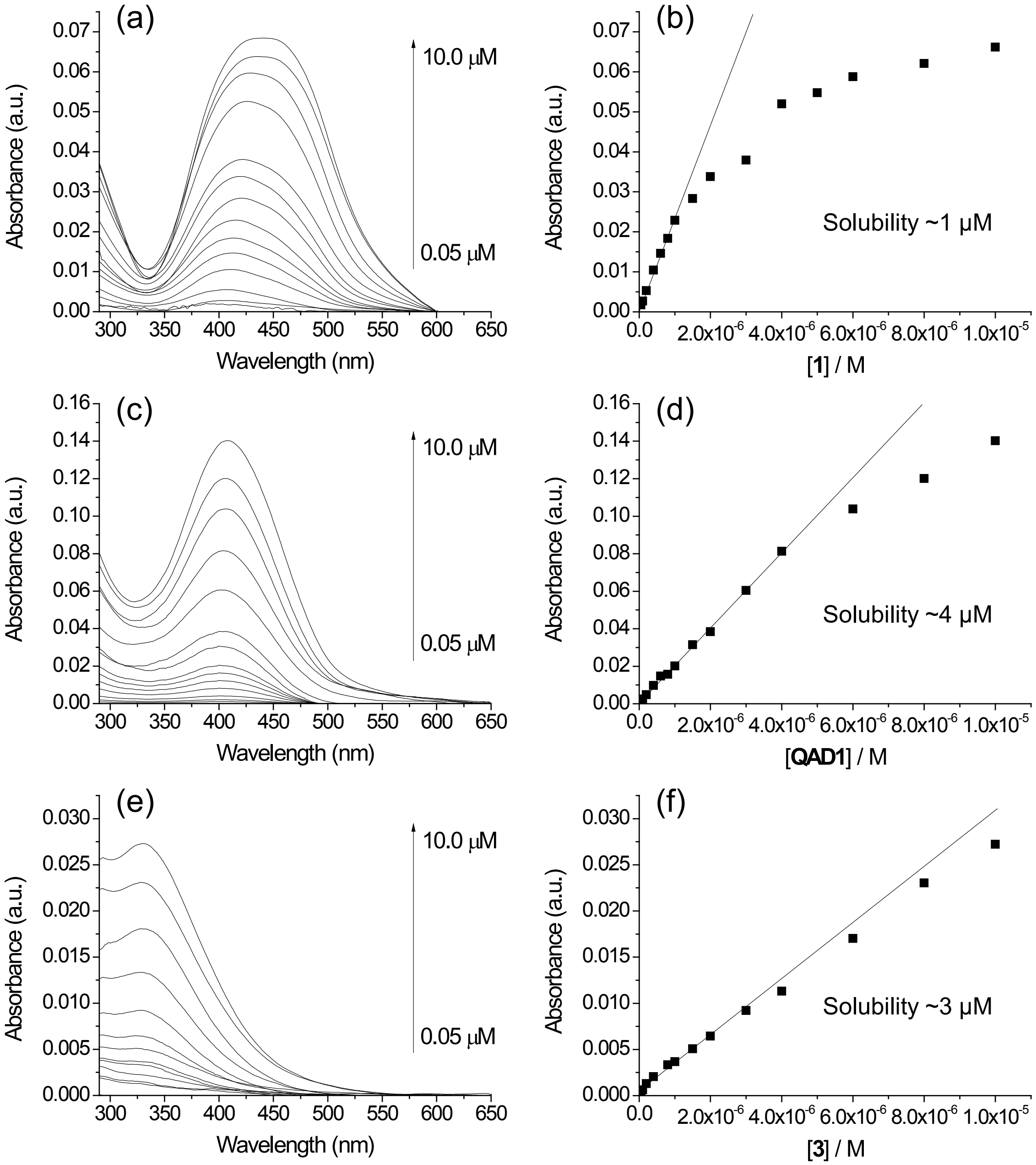

Fig. S1 (a,c,e) One-photon absorption spectra and (b,d,f) plot of intensity against probe concentration for (a,b) **1**, (c,d) **QAD1** and (e,f) **3** in PBS buffer (10mM, pH 7.4).

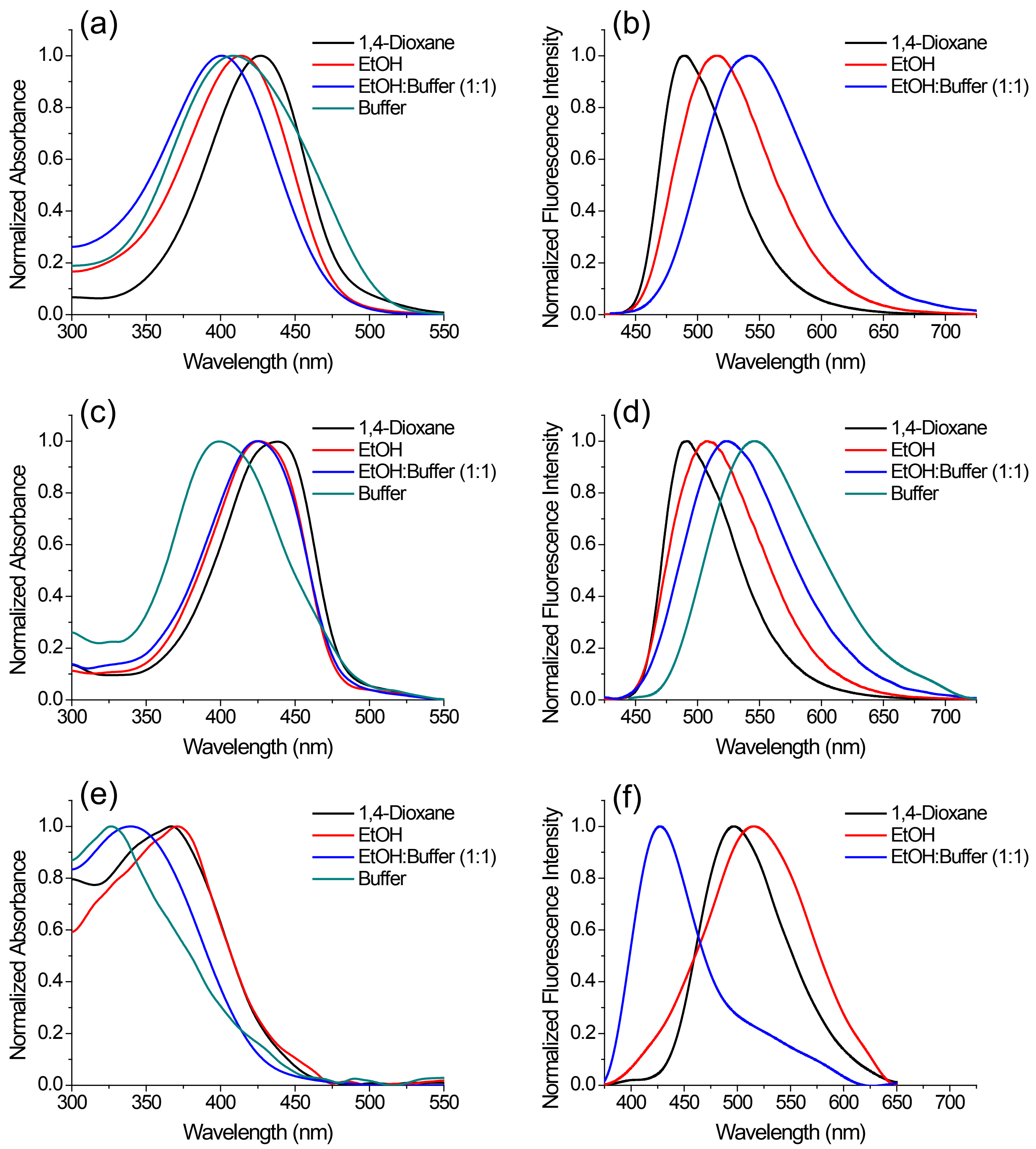

Fig. S2 (a, c, e) Normalized absorption and (b, d, f) emission spectra of (a, b) **1**, (c, d) **QAD1** and (e, f) **3** and in 1,4-dioxane, EtOH, EtOH: PBS (v/v, 1:1) and PBS buffer (10 mM, pH 7.4).

Table S1. Photophysical data for **1**, **QAD1** and **3** in various solvents.

Probe	Solvent (E_T^N)[a]	$\lambda_{max}^{(1)}$ [b]	λ_{max}^{fl} [c]	Φ[d]	$\lambda_{max}^{(2)}$ [e]	δ[f]	$\Phi\delta$[g]
**1**	1,4-Dioxane (0.164)	427	489	0.97	-	-	-
	EtOH (0.654)	413	515	0.50	750	540	270
	EtOH:PBS (1:1)	401	538	0.06	-	-	-
	PBS (1.00)[h]	417	ND[i]	ND[i]	-	-	-
**QAD1**	1,4-Dioxane (0.164)	438	490	1.00	-	-	-
	EtOH (0.654)	426	508	0.73	750	575	420
	EtOH:PBS (1:1)	424	523	0.10	-	-	-
	PBS (1.00)[h]	407	546	0.01	-	-	-
**3**	1,4-Dioxane (0.164)	367	495	0.28	-	-	-
	EtOH (0.654)	371	521	0.03	720	80	2
	EtOH:PBS (1:1)	339	422	0.04	-	-	-
	PBS (1.00)[h]	326	ND[i]	ND[i]	-	-	-

[a] The numbers in the parenthesis are normalized empirical parameter of solvent polarity.[3] [b] λ_{max} of the one-photon absorption spectra in nm. [c] λ_{max} of the one-photon emission spectra in nm. [d] Fluorescence quantum yield. The uncertainty is ± 10 %. [e] λ_{max} of the two-photon excitation spectra in nm. [f] The peak two-photon absorption cross-section in 10^{-50} cm^4s/photon (GM). [g] The peak two-photon action cross-section in 10^{-50} cm^4s/photon. [h] PBS buffer (10mM, pH 7.4). The E_T^N value is for water. [i] Not determined. The one- and two-photon excited fluorescence signals were too small to determine the values.

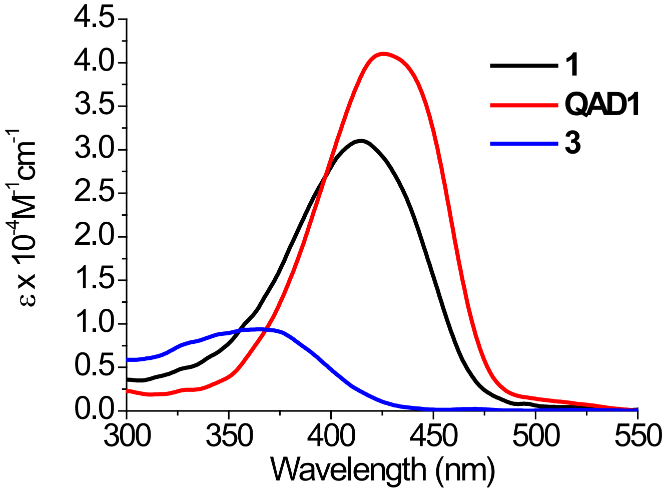

Fig. S3 Molar absorptivity spectra of **1**, **QAD1** and **3** (1 μM) acquired in EtOH.

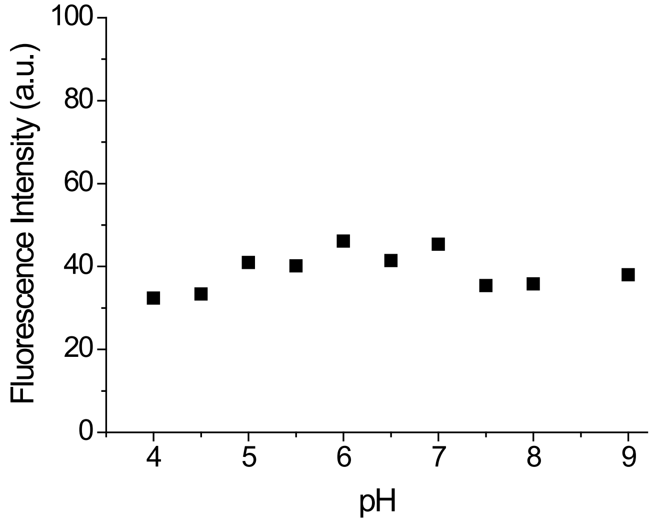

Fig. S4 Effect of pH for **QAD1** in universal buffer (0.1 M citric acid, 0.1 M KH_2PO_4, 0.1 M $Na_2B_4O_7$, 0.1 M Tris, 0.1 M KCl). The excitation wavelength was 407 nm.

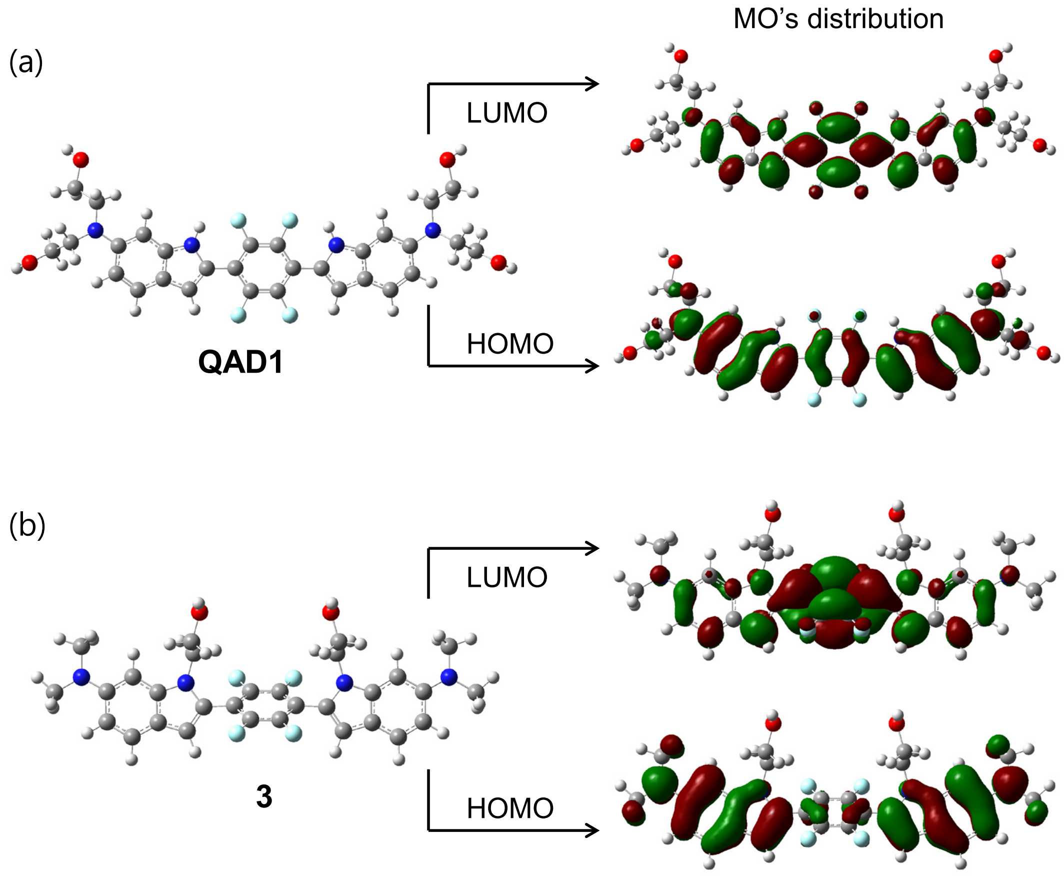

Fig. S5 DFT (Density Functional Theory) optimized geometries and MO's distribution of (a) **QAD1** and (b) **3** in EtOH.

Table S2. DFT (Density functional theory) calculation of **QAD1** and **3** in EtOH.

Probe	*isomer*	Relative energy (kJ/mol)	HOMO-LUMO energy (eV)	Oscillator strength
**QAD1**	*cis*	0.09	2.56	1.91
	trans	0	2.56	1.92
**3**	*cis*	0.44	2.67	0.98
	trans	0	2.66	0.97

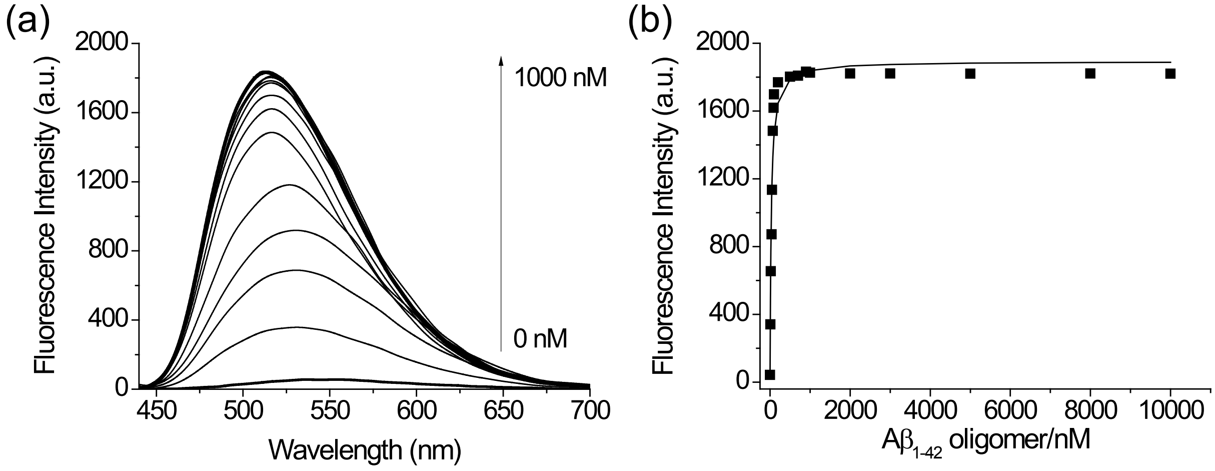

Fig S6. (a) Change in emission intensity and (b) the fluorescence titration curve for the complexation of **QAD1** (1 μM) with Aβ_{1-42} oligomer (0-10 μM) in PBS buffer (10 mM, pH 7.4). The calculated value is represented by solid line. The excitation wavelength was 407 nm and the fluorescence intensity was measured at 517 nm.

Table S3. Complexation Study of **QAD1** with Aβ_{1-42} aggregates and oligomer.[a,b]

Probe	$\lambda_{max}^{(1)}$ [c]	λ_{max}^{fl} [d]	Φ [e]	K_d [f]	FEF [g]
**QAD1**	407	546	0.01	16.2 ± 1.8 (nM)	85
**QAD1** + Aβ_{1-42} aggregates	455	510	0.43		
**QAD1**	407	546	0.01	21.5 ± 1.7 (nM)	43.2
**QAD1** + Aβ_{1-42} oligomer	441	517	0.27		

[a] See Scheme 1 in the text for the chemical structure. [b] All data measured in PBS buffer (10 mM, pH 7.4) in the absence and presence of 10 μM Aβ_{1-42} aggregated and oligomer. [c] λ_{max} of the one-photon absorption spectra in nm. [d] λ_{max} of the one-photon emission spectra in nm. [e] Fluorescence quantum yield. The uncertainty is ±10%. [f] Dissociation constants measured by one-photon processes in the presence of Aβ_{1-42} aggregated (0-10 μM) and Aβ_{1-42} oligomer (0-10 μM). The uncertainty is ±10%. [g] Fluorescence enhancement factor, $(F-F_{min})/F_{min}$.

Table S4. Photophysical data of two-photon imaging probes for Aβ plaques.[a]

Probe	$\lambda_{max}^{(1)}$ (10^{-4}) [b]	λ_{max}^{fl} [c]	Φ[d]	$\lambda_{max}^{(2)}$ [e]	δ[f]	$\Phi\delta$[g]	log P_{oct} [h]	K_d [i]	FEF [j]
**QAD1**	426 (4.09)	508	0.73	750	575	420	3.4	16.2[k]	85
**PIB**[4]	353	417	1.00	740	40	40	1.2	41[l]	-
**MeO-X04**[4]	372	444	1.00	720	75	75	2.6	N.D.[m]	N.D.[m]
**SAD1**[4]	370	465	1.00	750	170	170	1.9	17[l]	-
**5**[5]	512 (1.67)	679	0.05	N.A.[n]	N.A.[n]	N.A.[n]	3.5[o]	44.6[p]	60

[a] All the measurements were performed in EtOH. [b] One-photon absorption spectra in nm. The numbers in parentheses are molar extinction coefficients in $M^{-1}cm^{-1}$. [c] One-photon emission spectra in nm. [d] Fluorescence quantum yield. The uncertainty is ± 10 %. [e] Two-photon excitation spectra in nm. [f] Two-photon absorption cross-section in 10^{-50} cm^4s/photon (GM). The uncertainty is ± 15 %. [g] Two-photon action cross section in 10^{-50} cm^4s/photon (GM). [h] Calculated value of lipophilicity measured in octanol. [i] Dissociation constants (nM) measured in the presence of $A\beta_{1-42}$ aggregated. [j] Fluorescence enhancement factor. [k] Measured in PBS buffer (10 mM, pH 7.4). [l] Measured in HBS-EP buffer (consisting of 0.01 mol/L HEPES, 0.15 mol/L NaCl, 3 mmol/L EDTA, 0.005% surfactant P20, pH 7.6). [m] Not determined. [n] Not available. [o] Calculated using ACDLab-ACDLog *P* software. [p] Measured in PBS buffer (10 mM, pH 7.4, containing 1 % DMSO).

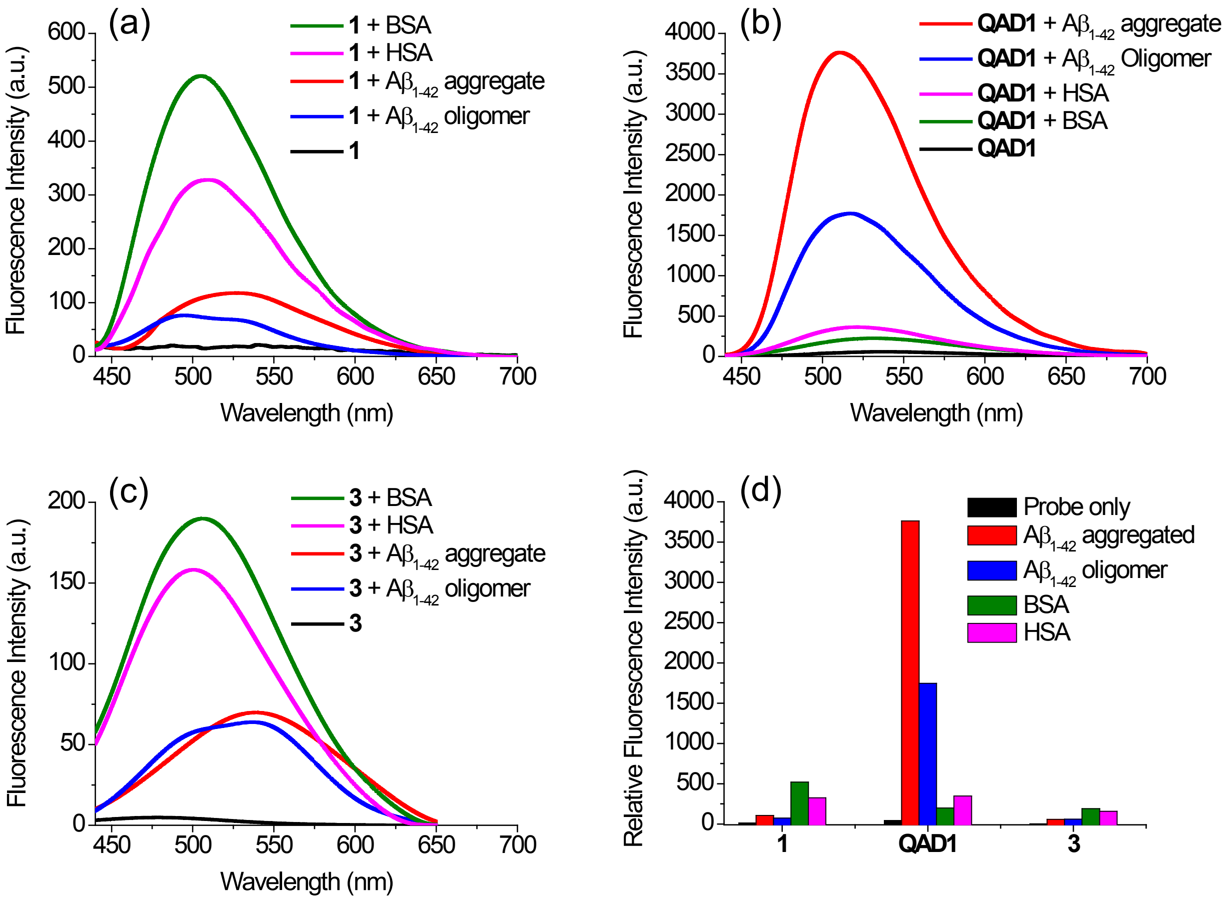

Fig. S7 The change in emission intensity (a) **1**, (b) **QAD1** and (c) **3** in presence of $A\beta_{1-42}$ aggregated, $A\beta_{1-42}$ oligomer, BSA and HSA in PBS buffer (10 mM, pH 7.4). (d) Relative fluorescence intensity upon addition of particular protein in PBS (10 mM, pH 7.4) measured at 504, 510 and 506 nm for **1**, **QAD1** and **3**, respectively. The excitation wavelengths were 417 nm for **1**, 407 nm for **QAD1** and 326 nm for **3**, respectively.

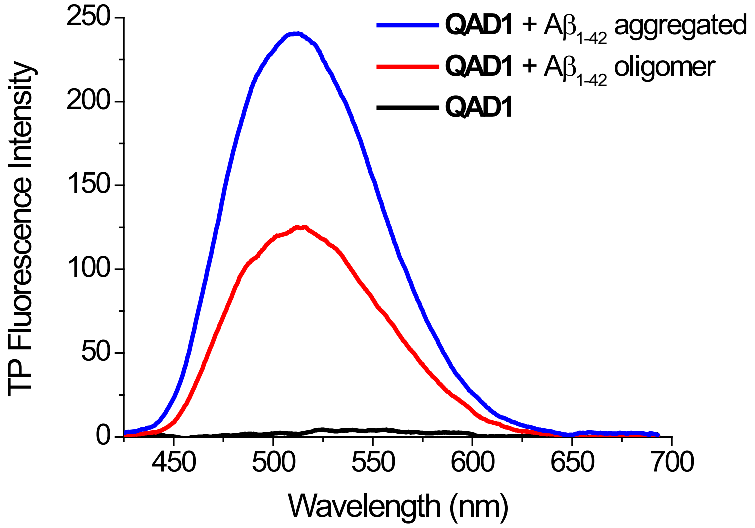

Fig. S8 Two-photon excited fluorescence response of 1 μM **QAD1** in the presence of Aβ_{1-42} aggregates (10 μM) and Aβ_{1-42} oligomer (10 μM) in PBS buffer (10 mM, pH 7.4). The excitation wavelength was 750 nm.

Octanol-water partition coefficient (log P_{oct}). Small aliquot (10 μL) of 20 mM probe solution in DMSO was added to a vial containing 5 mL *n*-octanol by using a micro syringe. To this solution, 5 mL of PBS buffer was added. The resulting mixture was stirred vigorously and kept in dark for 1 day. The concentrations of probe in each layer were determined by the UV-Vis absorbance with their molar extinction coefficients as shown in Table S4. The log P_{oct} value was calculated by using log P_{oct} = log [probe]$_{oct}$ – log [probe]$_{PBS}$: where the [probe]$_{oct}$ and [probe]$_{PBS}$ are the concentrations of the probe in *n*-octanol and PBS, respectively. The log P_{oct} values for each probe was summarized in Table S4.

Table S5. Molar extinction coefficients and logP_{oct} for listed probes in *n*-octanol and PBS.

Probe	Solvent	ε ($10^{-4}M^{-1}cm^{-1}$)	log P_{oct}
**1**	*n*-octanol	3.68	5.22
	PBS	1.87	
**QAD1**	*n*-octanol	4.12	3.42
	PBS	2.25	
**3**	*n*-octanol	0.496	5.14
	PBS	0.362	

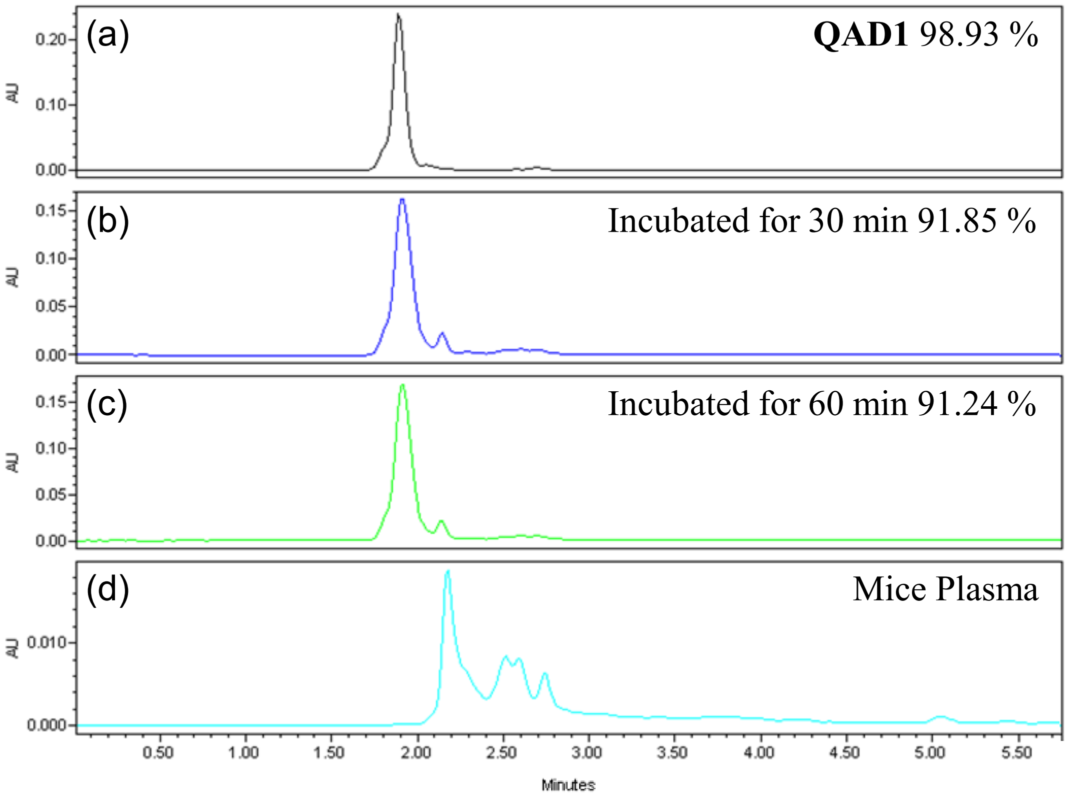

Fig. S9 The HPLC profile of **QAD1** (a) before and (b,c) after incubation with mice plasma for (b) 30 and (c) 60 min. (d) The HPLC profile of mice plasma.

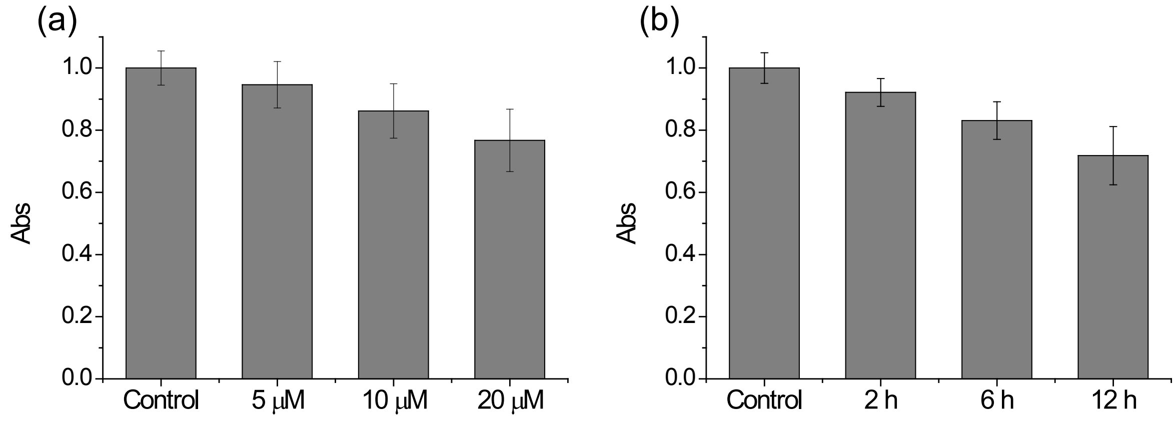

Fig. S10 Viability of SH-SY5Y cells in the presence of **QAD1** as measured by using MTS assay. The cells were incubated with (a) 0–20 μM **QAD1** for 12 h and (b) 20 μM **QAD1** for 2, 6 and 12 h. (n = 6)

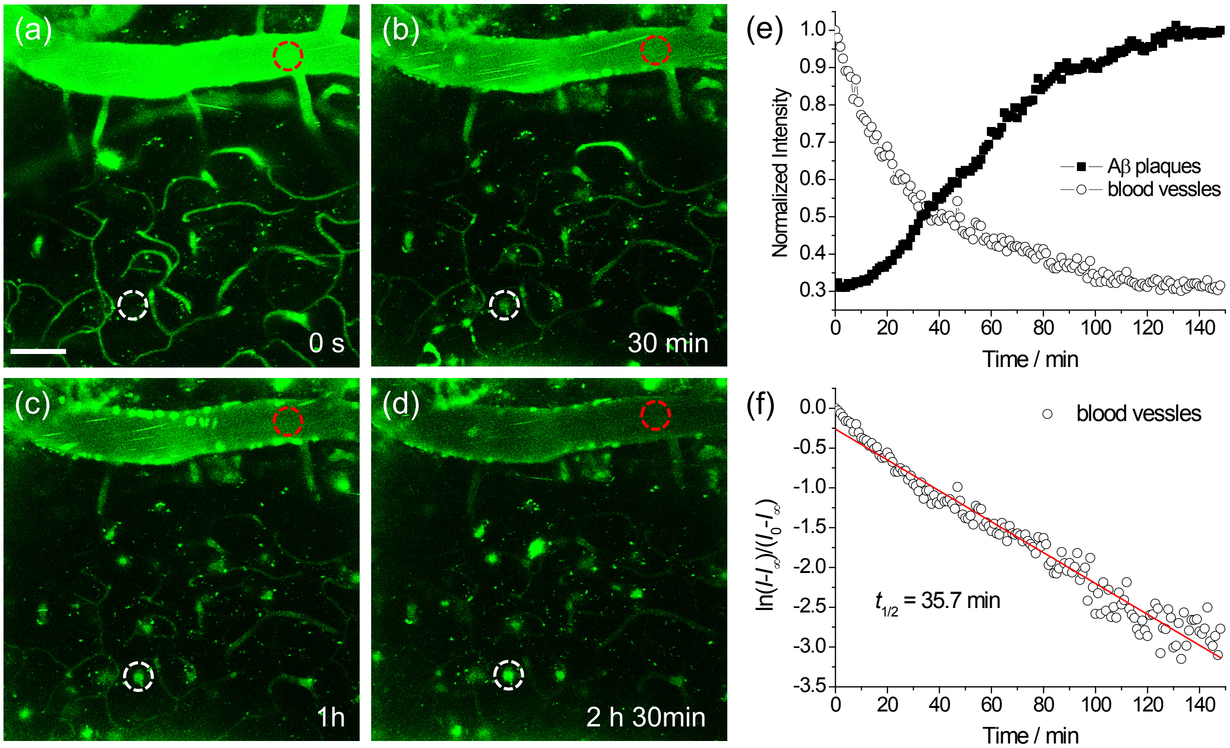

Fig. S11 *In Vivo* TPM images of the frontal cortex of transgenic 5XFAD mice at (a) 0, (b) 30, (c) 60 and (d) 150 min after i.v. injection of **QAD1** (10mg/Kg). Scale bar: 50 μm. (e) Two-photon excited fluorescence intensity (normalized) as a function of time acquired from the blood vessels (red circle in Fig S11 a-d) and the plaque (white circle in Fig S11 a-d). (f) Decay rates of the TPEF in the blood vessels (red circle). The slopes of these plots are the 1st order decay constant. Half-life ($t_{1/2}$) were calculated by using the relationship, $t_{1/2} = 0.693/k$.

^{1}H-NMR, ^{13}C-NMR and HRMS of **1**, **QAD1** and **3.**

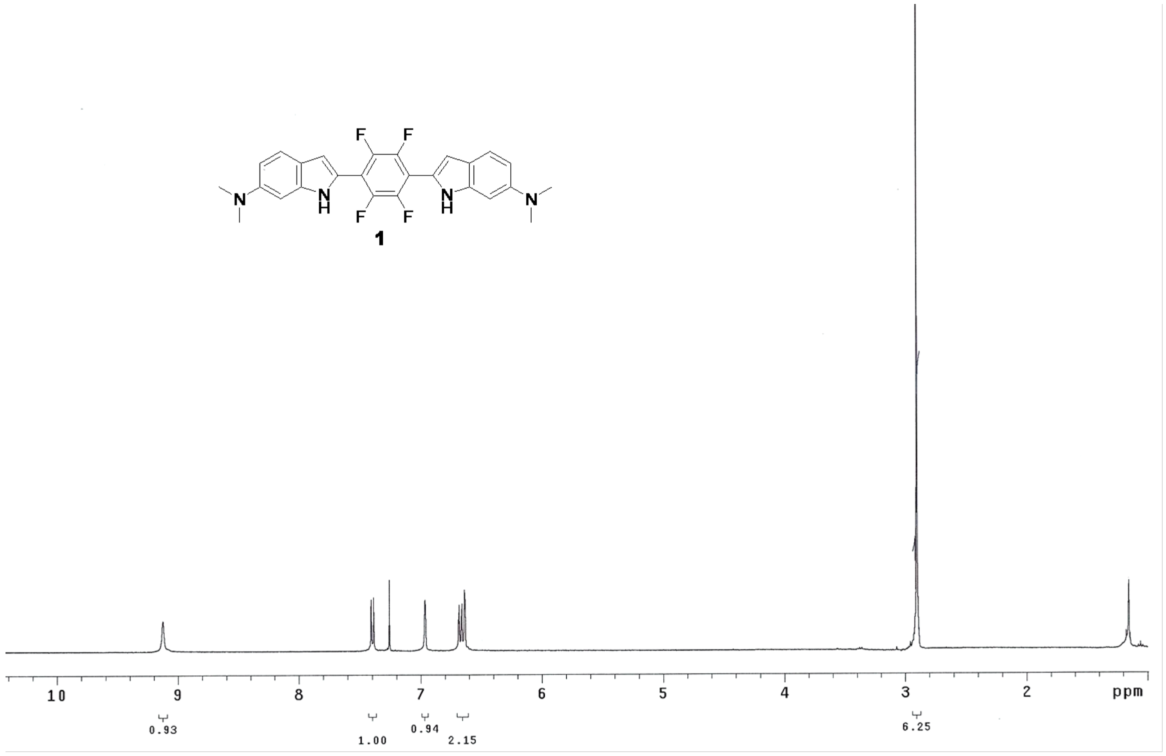

Fig. S12 ^{1}H-NMR spectrum (400 MHz) of **1** in $CDCl_3$.

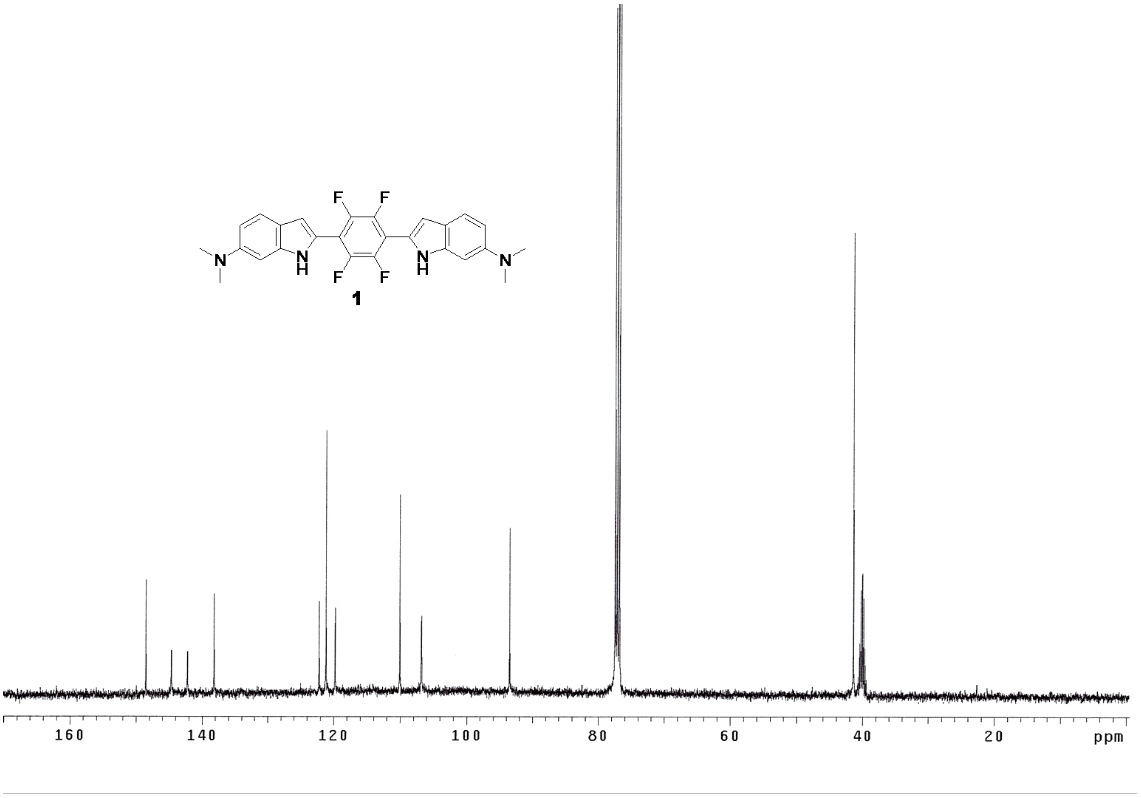

Fig. S13 ^{13}C-NMR spectrum (100 MHz) of **1** in $CDCl_3$ containing 2 drops of *d*$_6$-DMSO.

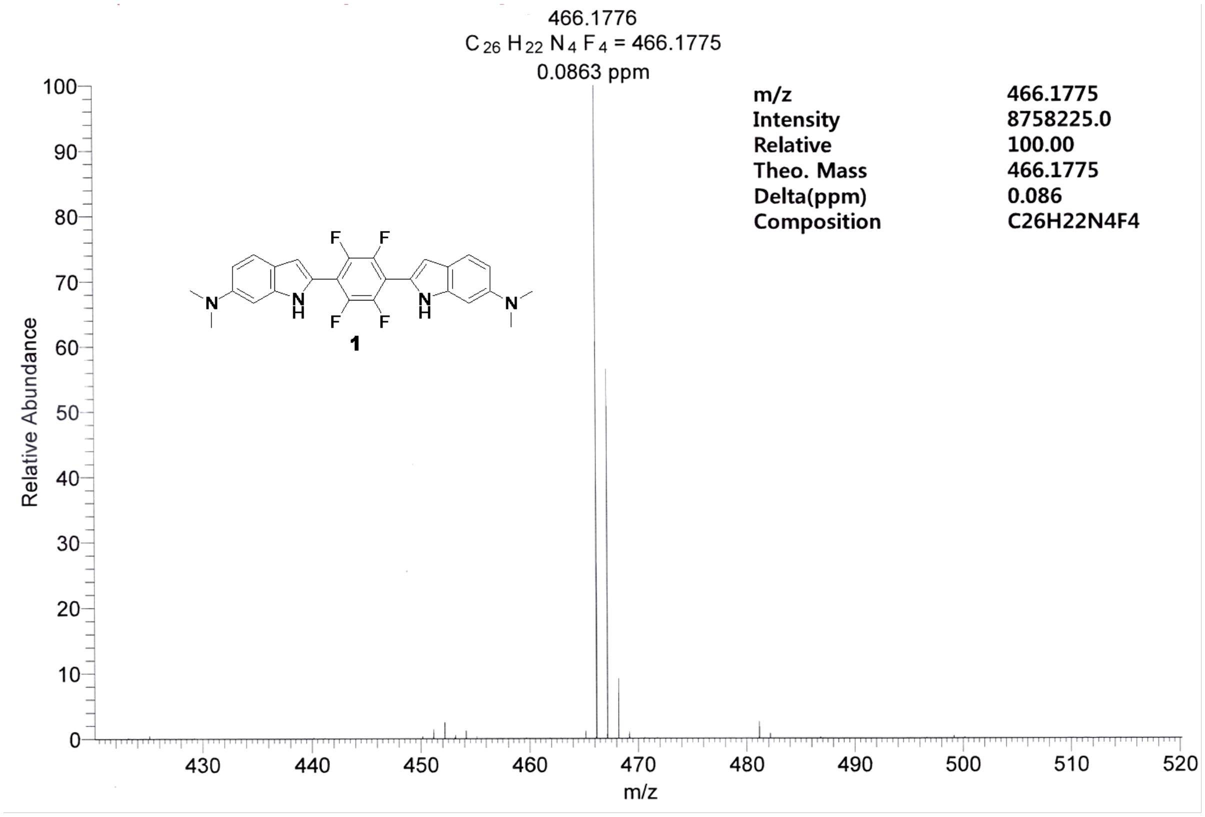

Fig. S14 HRMS spectrum of **1.**

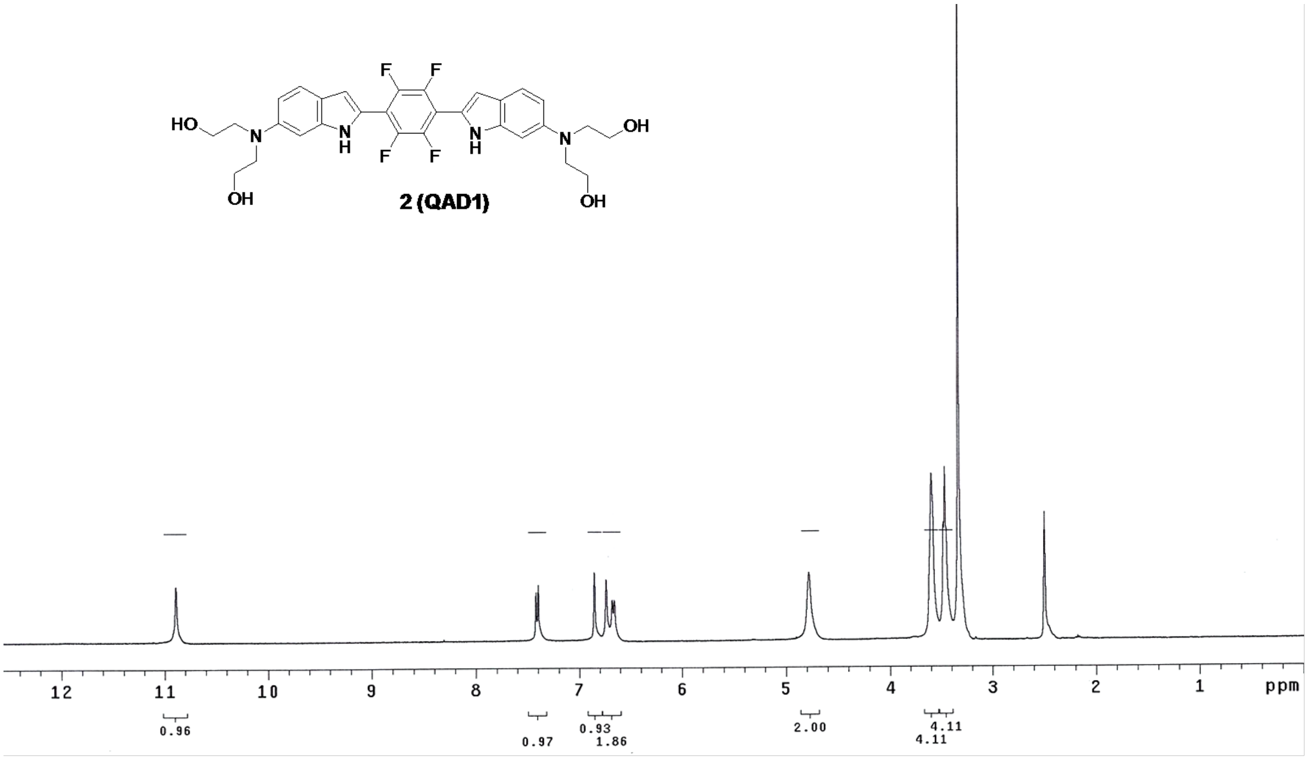

Fig. S15 ^{1}H-NMR spectrum (400 MHz) of **2** (**QAD1**) in d_6-DMSO.

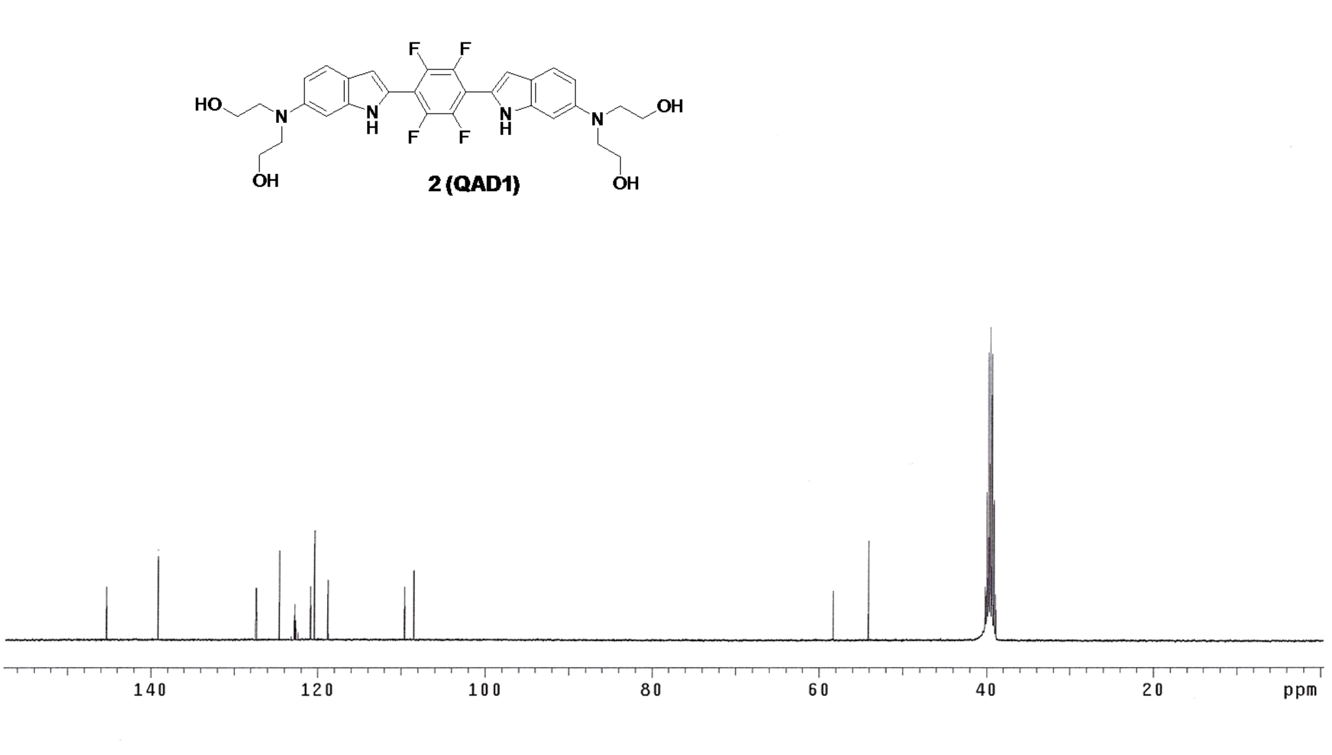

Fig. S16 ^{13}C-NMR spectrum (100 MHz) of **2** (**QAD1**) in d_6-DMSO.

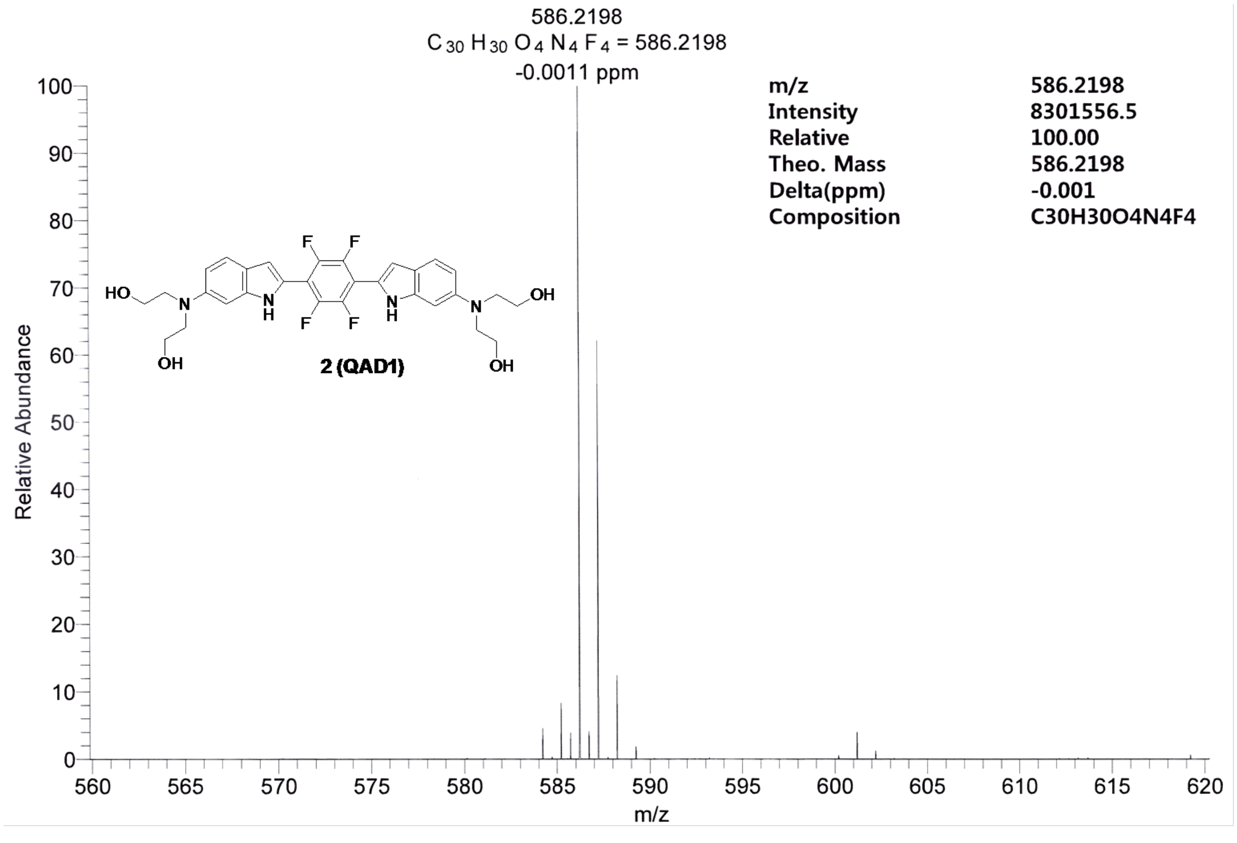

Fig. S17 HRMS spectrum of **2** (**QAD1**).

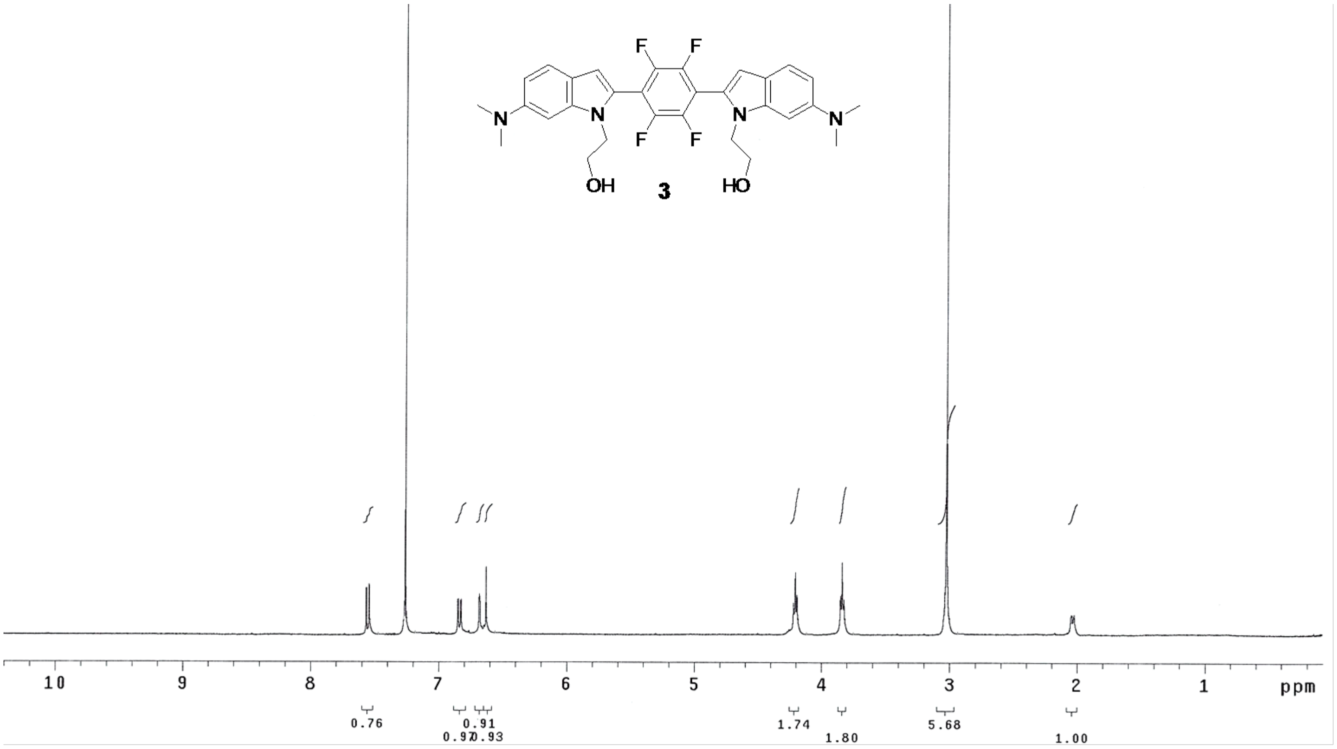

Fig. S18 ^{1}H-NMR spectrum (400 MHz) of **3** in $CDCl_3$.

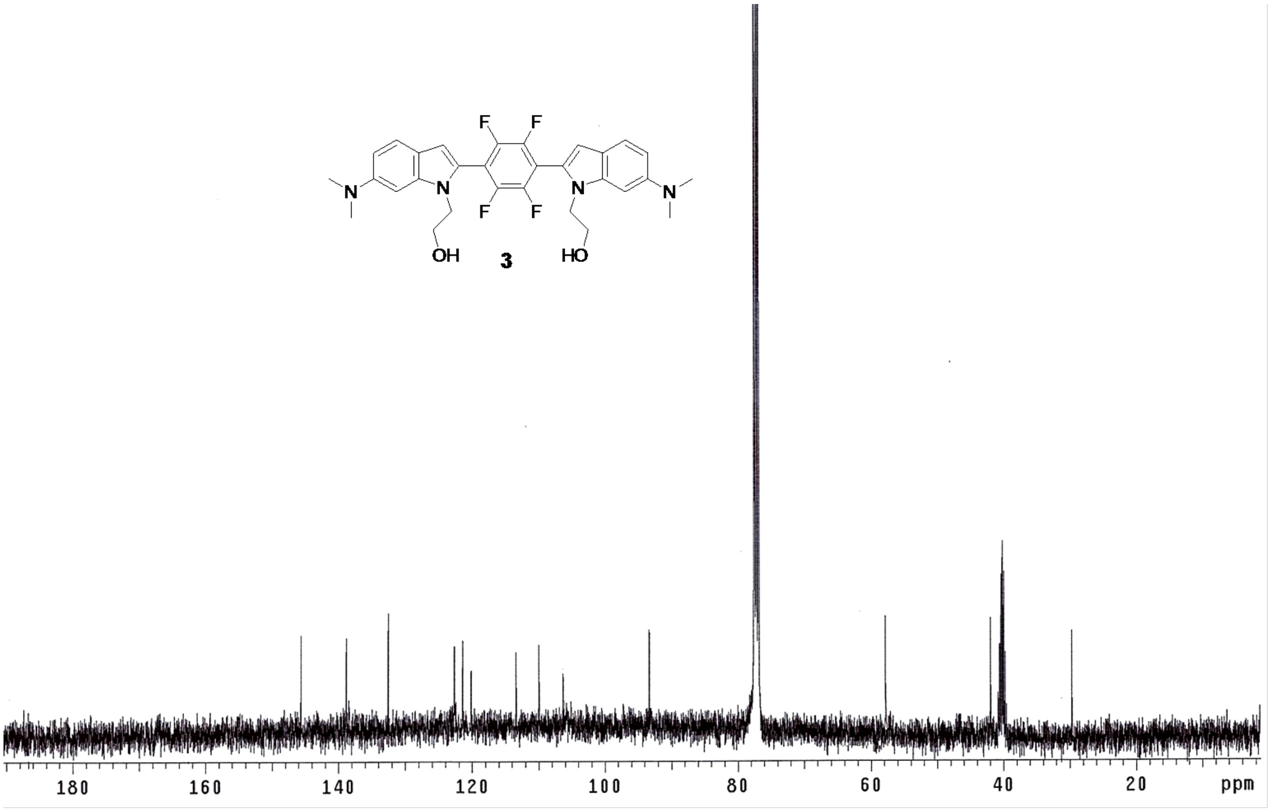

Fig. S19 ^{13}C-NMR spectrum (100 MHz) of **3** in $CDCl_3$ containing 2 drops of d_6-DMSO.

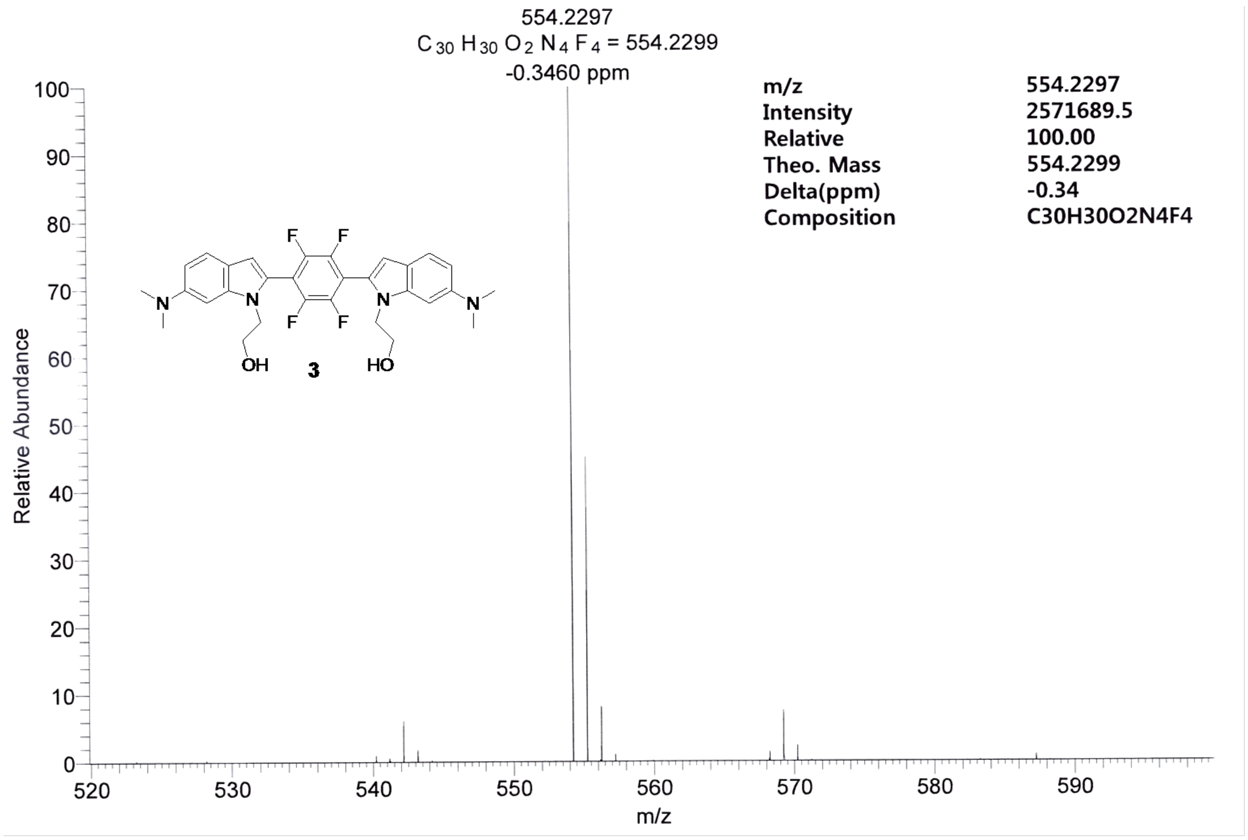

Fig. S20 HRMS spectrum of **3.**

References

1. F. C. Krebs, T. Jensen, *J. Fluorine Chem.* 2003, **120**, 77.
2. H. M. Kim, H. J. Choo, S. Y. Jung, Y. G. Ko, W. H. Park, S. J. Jeon, C. H. Kim, T. Joo, B. R. Cho, *ChemBioChem* 2007, **8**, 553.
3. C. Reichardt, *Chem. Rev.* 1994, **94**, 2319.
4. C. H. Heo, K. H. Kim, H. J. Kim, S. H. Baik, H. Song, Y. S. Kim, J. Lee, I. Mook-jung, H. M. Kim, *Chem. Commun.* 2013, **49**, 1303.
5. D. Kim, H. Moon, S. H. Baik, S. Singha, Y. W. Jun, T. Wang, K. H. Kim, B. S. Park, J. Jung, I. Mook-Jung, K. H. Ahn, *J. Am. Chem. Soc.* 2015, **137**, 6781.
